# Mechanobiology as a tool for addressing the genotype-to-phenotype problem in microbiology

**DOI:** 10.1063/5.0142121

**Published:** 2023-05-12

**Authors:** Merrill E. Asp, Minh-Tri Ho Thanh, Subarna Dutta, Jessica A. Comstock, Roy D. Welch, Alison E. Patteson

**Affiliations:** 1 Physics Department, Syracuse University, Syracuse, New York 13244, USA; 2 Biology Department, Syracuse University, Syracuse, New York 13244, USA; 3 BioInspired Institute, Syracuse University, Syracuse, New York 13244, USA

## Abstract

The central hypothesis of the genotype–phenotype relationship is that the phenotype of a developing organism (i.e., its set of observable attributes) depends on its genome and the environment. However, as we learn more about the genetics and biochemistry of living systems, our understanding does not fully extend to the complex multiscale nature of how cells move, interact, and organize; this gap in understanding is referred to as the genotype-to-phenotype problem. The physics of soft matter sets the background on which living organisms evolved, and the cell environment is a strong determinant of cell phenotype. This inevitably leads to challenges as the full function of many genes, and the diversity of cellular behaviors cannot be assessed without wide screens of environmental conditions. Cellular mechanobiology is an emerging field that provides methodologies to understand how cells integrate chemical and physical environmental stress and signals, and how they are transduced to control cell function. Biofilm forming bacteria represent an attractive model because they are fast growing, genetically malleable and can display sophisticated self-organizing developmental behaviors similar to those found in higher organisms. Here, we propose mechanobiology as a new area of study in prokaryotic systems and describe its potential for unveiling new links between an organism's genome and phenome.

## I. INTRODUCTION

**FIG. 1.**
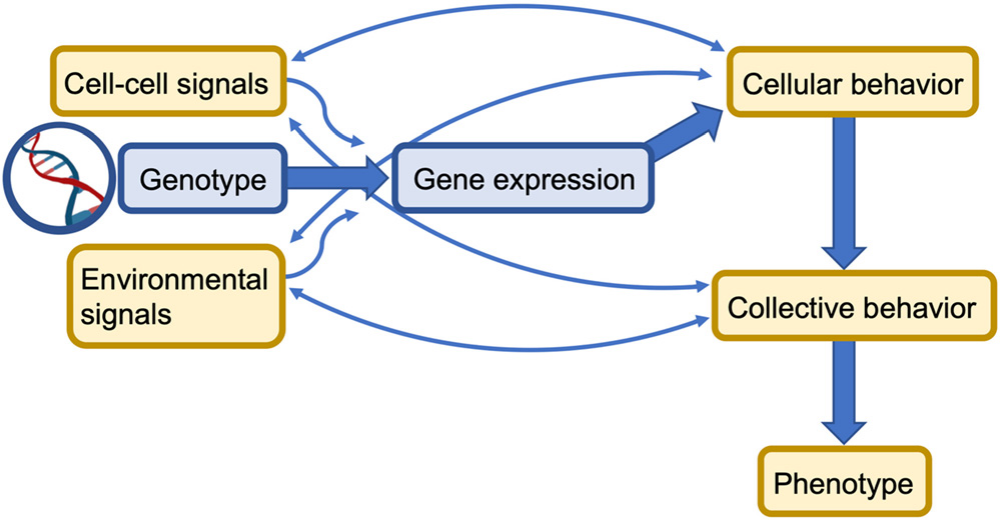
Causal web of the genotype-to-phenotype problem: genetic instructions stored as DNA are expressed as phenotypes of collective living systems, but this occurs through a cyclical network of cause and effect. Specific genes are expressed either to run necessary cellular functions or in response to signals from outside the cell. These changes in gene expression alter the behavior of individual cells and may also produce signaling molecules that influence the gene expression of neighboring cells. These collective changes in behavior can, in turn, influence the quantity and characteristics of the cell-to-cell signals—for instance by changing cell motility—or even alter the local environment—for instance by excreting extracellular matrix compounds. These feedback signals allow the collective behavior to evolve over time into the resultant, emerging phenotype.

Cells with identical genomes self-organize into distinct and diverse tissues with different properties and functions.^1^ The cell genome encodes all the proteins and molecular machinery a cell may synthesize and express. Traditionally, gene function is deduced by examining the impact of its mutation on cell and tissue morphology and development. A complete description of development was, thus, once thought to be found in studying the genome in more depth and rigor. However, even when we know an organism's full genome, it is often not sufficient to predict cellular behaviors. In biology, this is referred to as the genotype-to-phenotype problem (Fig. 1). A phenotype is the observable characteristics of a living system, and it is understood that an organism's phenotype is a function of its genome and its environment. Plants grow toward light, fungi form spores when starved, and bacteria self-organize into multicellular biofilms when in contact with a surface. External physical and chemical environmental cues shape developmental decisions.

Cells have sensors that detect chemical and/or physical signals. Chemical signal sensors are typically molecular receptors that are part of signaling pathways that sense and respond to specific chemical stimulants. A well-studied example of such a pathway controls chemotaxis, in which bacteria display directed motility toward or away from a chemical gradient. The chemotactic response to chemical signals is well characterized and understood at the cellular and molecular level. In contrast, the cellular and molecular response to physical stimuli is far less well understood.^2^ The best described physical sensors are those that detect mechanical features of the extracellular environment, and these typically rely on the coupled motion of a cellular organelle with the environment. Cells transduce these mechanical cues into biochemical signals to adapt their behavior, a process termed mechanotransduction. Examples include the deflection of primary cilia in ear cells to hear sound^3^ and membrane-based sensors that detect pressure gradients across the cell membrane.^4^

**FIG. 2.**
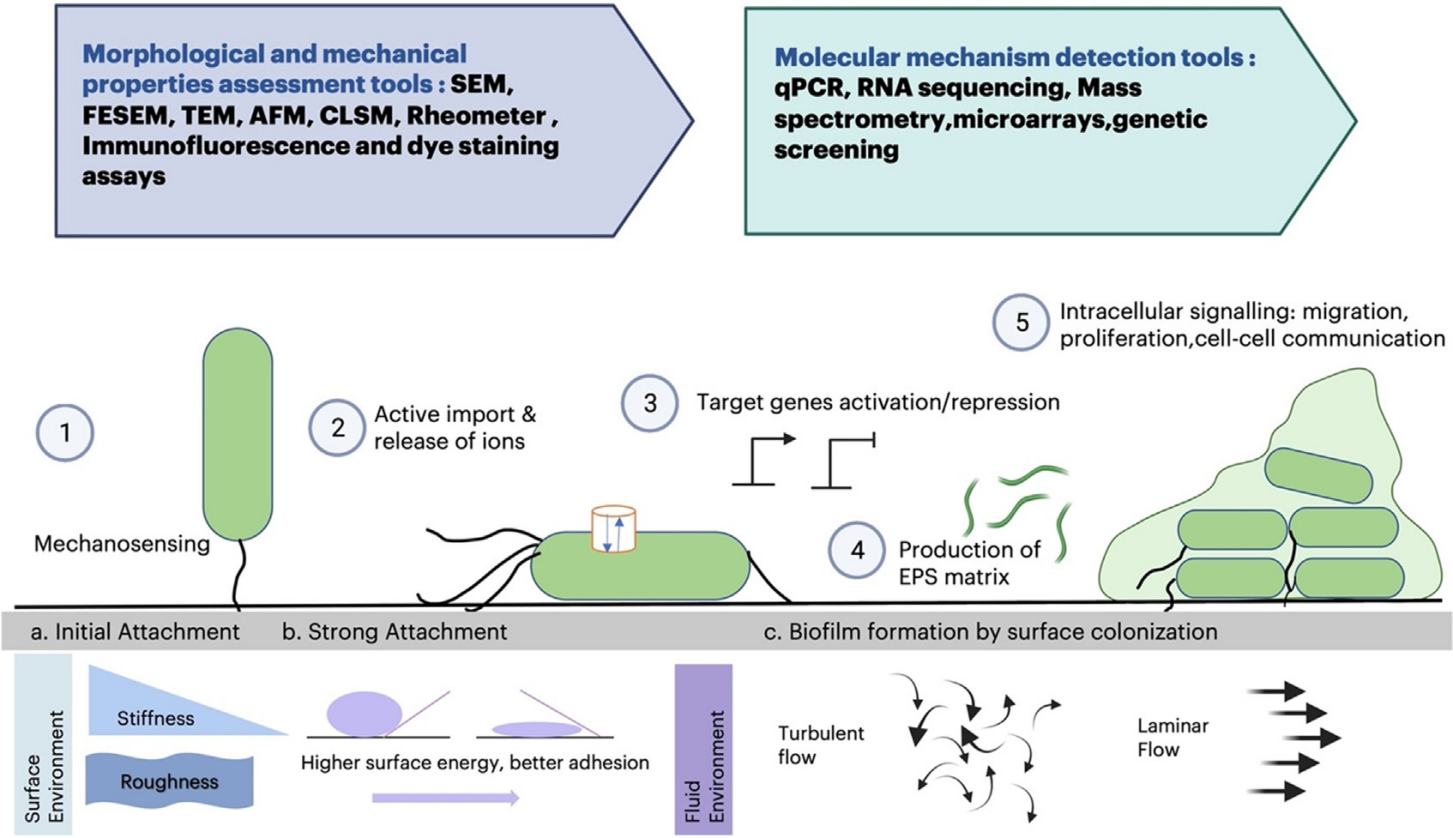
Mechanistic insights into the formation of bacterial biofilm and tools of characterization. The blue arrow denotes tools for morphological and mechanical properties assessment and the green arrow depicts tools for molecular mechanism detection. Numbers 1–5 depict sequence of events in the multicellular formation of bacteria colonies.^1^ The first step is bacteria making contact with a surface. Mechanosensing involves the transduction of mechanical input (surface contact) to a bacterial response and activation of distinct cellular machineries. Upon surface contact, biofilm formation commences by active import and release of ions,^2^ target gene activation and repression,^3^ production of EPS matrix, and^4^ intracellular signaling, migration, proliferation, and cell–cell communication. Environmental features that impact multicellular bacteria pattern formation include surface stiffness, roughness, adhesion, and surface tension, as well as ambient fluid flow that may be turbulent or laminar.

**FIG. 3.**
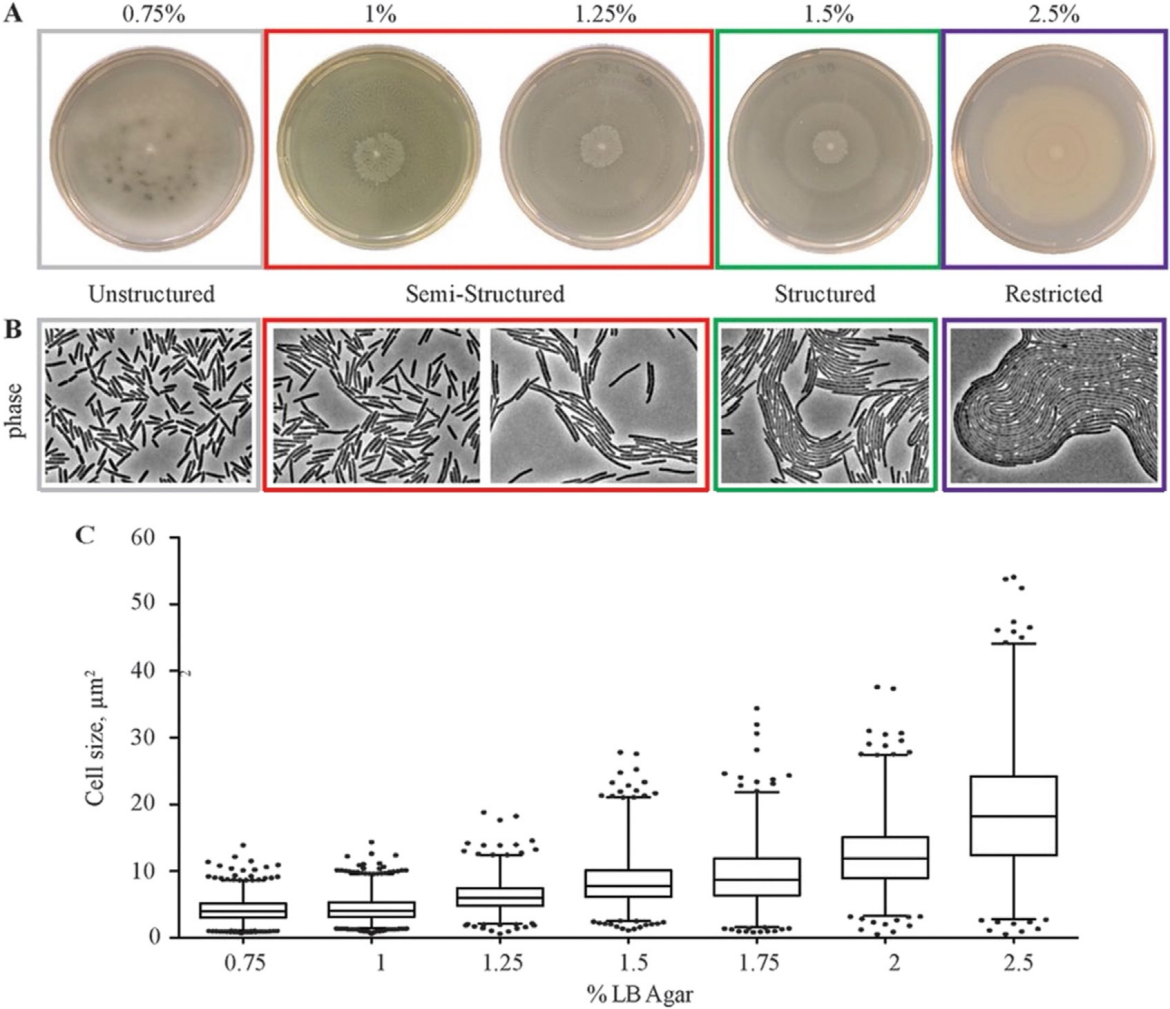
Effects of agar concentration on single cell and multicellular colony morphology. (a) The bacterium *Proteus mirabilis* forms swarming colonies on nutrient-rich low-concentration agar (1.5% agar) surfaces. Increasing agar changes the population morphology with the emergence of structured colony terraces and restricted growth above 2.5% agar. (b) Phase contrast images of cells as a function of increasing agar concentration. (c) Cell length increases with increasing agar concentration. Figure adopted from Ref. 71. Reproduced with permission from Little *et al.*, J. Bacteriol. **201**, e00726-00718 (2019). Copyright 2019 Authors, licensed under a Creative Commons Attribution (CC BY) license.

In the last 20 years, the field of mechanobiology has emerged to study how mechanics govern cell phenotypes. Most work has focused on human cells and animal models, as leading motivation has been the observation that cells behave differently in tissues of different stiffness,^5^ a critical component of diagnosing disease such as cancer and fibrosis and which cannot be explained purely in biochemical terms. New work is also highlighting how bacteria, which also can exhibit many features of collective cell morphogenesis and development, sense and respond to the mechanical features of their environment (Fig. 2). Here, we propose bacteria systems as an attractive model for identifying fundamental mechanisms of mechanosensing and highlight its relevance to addressing the genotype-to-phenotype problem. Bacteria biofilms can display a number of multicellular behaviors similar to those found in higher organisms such as swarming,^6–8^ predation,^9,10^ and aggregation.^11–13^ Studies of bacterial mechanosensing have been limited in number and scope, in part because of size and structural differences between bacterial and mammalian cells. Thus, a number of mammalian experimental tools cannot be directly applied to bacterial systems. However, bacterial and mammalian cells do share some important measurable attributes. Like mammalian cells, bacteria, can respond to physical stress by changing shape,^15,16^ migrating,^17–19^ differentiating,^20,21^ and altering gene expression^22,23^ (Fig. 3). Many mammalian experimental tools can be modified to apply to bacterial systems. In this article, we briefly review the mechanics of bacteria motility and associated protein activity, summarize recent advances in the field of bacteria mechanosensing, highlight the promise of mechanosensing techniques as a tool for the genotype-to-phenotype problem, and outline current challenges to the field.

## II. BACTERIA MOTILITY MODES AND COLONY GROWTH

Bacteria have a variety of mechanisms for generating force and motility both at the individual and collective level.^14,15^ Swimming bacteria, such as *Escherichia coli*, move by thin rotating helical filaments called flagella. The bacterial flagellum is a biological analog of a mechanical motor, complete with rotor and stator components. Flagella can also respond to changes in the physical environment: the motor runs at near-constant torque, such that the flagella rotation rates slow down when bacteria encounter more viscous fluids.^16^ While the bacteria flagella motor has long thought to be a stable structure within the cell, new work is revealing that its structure is dynamic. The motor proteins display transient binding kinetics,^17^ and recent studies with tethered cells indicate more motor components are recruited to the flagella motor in response to changes in mechanical load,^18^ suggesting a mechanosensing role of the bacteria flagella.

Swimming describes the motility of individual cells in liquid, but flagella can also move bacteria across surfaces in a form of motility called swarming. Bacterial swarming behavior is the fastest known mode of surface expansion.^6,19^ Swarming motility is driven by hyper-flagellated cells and seems to be narrowly conserved but is observed in both gram-negative (e.g., *E. coli*, *Serratia marcescens*) and gram-positive bacteria (e.g., *Bacillus subtilis*).^6^ Swarming is most common on soft substrates with high nutrient availability, highlighting the role of environmental conditions in eliciting distinct phenotypic transitions.

Flagella are one of several appendages that can power bacterial surface motility. For example, type IV pili (T4P) are long (micrometer) thin (nanometer) filaments that drive a form of motility called twitching.^20^ The twitching mechanism is similar to a winch in that a T4P will pull a cell across a surface by its ATP-powered extension, attachment, and retraction.^21^ A different form of surface motility is called gliding, which has been most extensively studied in *Myxococcus xanthus*. Gliding motility involves the formation of focal adhesion complexes elastically coupled to the cell's substrate and connected to the rotation of a cytoskeletal helix, driving cells forward like a corkscrew.^22^ Experiments using force-clamps estimated a force of 12 pN per focal adhesion node, which at approximately five focal adhesion sites per bacteria cell generates approximately 60 pN of force per cell, which is the same order of force estimated for twitching.^22^

Many surface-dwelling bacteria exist in dense populations, wherein interactions between cells can enable forms of motility not observed by cells in isolation. For example, colonies of cells can move on surfaces by sliding, a common form of collective expansion driven by pushing forces of dividing cells. This form of motion does not require active motor appendages and is accelerated by the production of surfactants that reduce surface tension.^23^ In *Staphylococcal* species, cell division in the absence of surfactants can drive a form of colony expansion called darting, in which cells are rapidly ejected from colony edges due to a build-up of pressure from cell division.^24^

In addition to sliding and darting, bacteria can coordinate phenotypic switches that allow for either collective slow-growing biofilm expansion or rapid surface expansion through swarming. The bacteria within a biofilm are encased in a protective self-secreted matrix of extracellular polymeric substance (EPS). During the process of biofilm formation, bacteria communicate through the exchange of signaling molecules. The mechanisms by which these signals are sent and received have as much impact on their function as the signals themselves. For example, in a mechanism called quorum sensing, the signal is produced inside each cell and secreted throughout the population, but the receptors reside on the outside of each cell, and its signal binding affinity is low enough that it activates only after the signal accumulates above a certain threshold outside the cells. Once reached, the biofilm cells undergo a change in phenotype that is synchronized by the signaling mechanism, thereby enabling a coordinated response, which involves the expression of hundreds of genes that promote cell differentiation and upregulation of many virulence factors. In addition to these kinds of chemical signals, a biofilm's EPS also contains a complex array of mechanical cues, in the form of viscoelastic materials; these are capable of both resisting and dissipating applied external forces, such as shear flow in a time-dependent manner. The viscoelasticity of the EPS largely dominates the mechanical properties of the biofilm itself, and the stiffness and strength of the EPS vary in different environments.^25^ The shear stiffness of different biofilms can vary significantly, from under 0.01 kPa to over 10 kPa,^26,27^ depending on the species, environmental conditions, and type of mechanical test. Biofilms appear capable of changing their mechanical properties in response to mechanical cues. For example, biofilms grown under higher shear are stronger than those grown under lower shear.^28^ Mechanotransduction, thus, occurs at both individual and population scales.

## III. MOLECULAR MECHANOTRANSDUCTION PATHWAYS

**FIG. 4.**
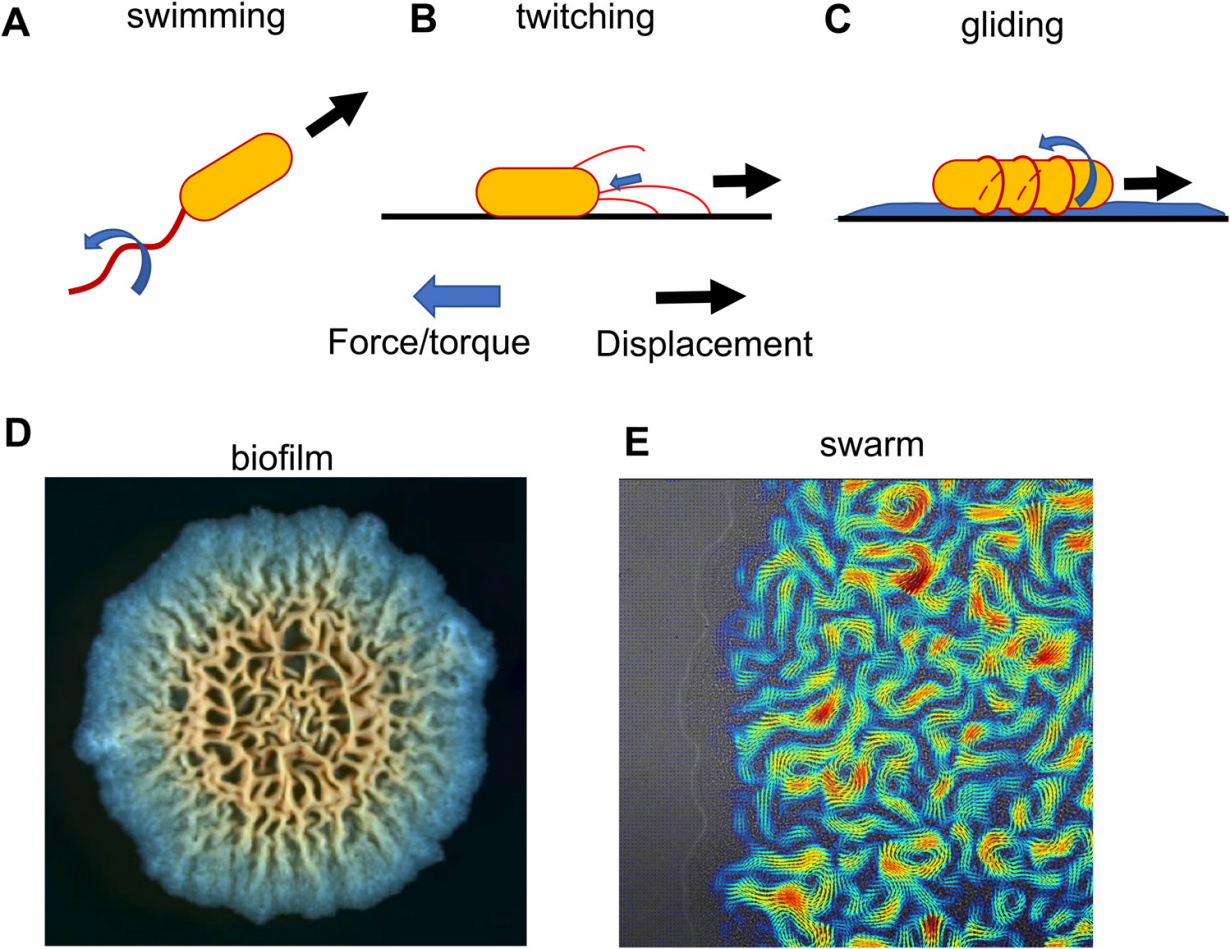
Bacterial motility. (a) Swimming motility powered by the rotation of bacterial flagella. (b) Twitching motility powered by the retraction of pili adhered to a solid surface. (c) Gliding motility, which in *Myxococcus xanthus* is powered by the rotation of a helical cytoskeletal track, moving the cell along a slime-coated surface, like a corkscrew tank. (d) Top-down image of *B. subtilis* colony. The biofilm is a more slowly growing colony than a bacterial swarm. In the biofilm, the bacteria produce extracellular polymeric substances, creating a semisolid matrix around the cells. Image adopted from Ref. 142. Reproduced with permission from Hou *et al.*, npj Biofilms Microbiomes **7**, 2 (2021). Copyright 2021 Authors, licensed under a Creative Commons Attribution (CC BY) license. (e) Swarming *Serratia marcescens* colony. Image shows the characteristic vortex patterns exhibited during swarming motility.

To sense physical features of their environment, cells use motorized machinery to transduce extracellular physical cues to biochemical signals in the cell interior (Fig. 4). Here, we briefly review how the transduction mechanism functions with respect to T4P, flagella, and other cell membrane protein complexes, with some focus on surface-sensing signaling pathways identified in a few pathogenic species.

In *Pseudomonas aeruginosa*, T4P has been identified with two distinct yet cooperative biochemical signaling pathways involving the secondary messengers cyclic-di-GMP (c-di-GMP) and cyclic AMP (cAMP), which promote a phenotypic switch to a biofilm state.^29–32^ Upon initial contact with attaching to a surface, T4P activity is kicked on by increased intracellular levels of c-di-GMP through its receptor FimW, which deploys the pilus to initiate adhesion.^33^ Over time, the activity of T4P stimulates cAMP production. The signaling molecule cAMP promotes the transcription of genes via the virulence factor regulator (Vfr), a transcription factor that initiates genes encoding secretion systems, components of the T4P, and important regulators of quorum sensing to initiate biofilm production.^29^ T4P is thought to directly regulate cAMP production through the two-component chemosensory system Chp. The Chp system is activated through the chemotaxis protein PilJ, which interacts directly with the major subunit of T4P, PilA.

Type IV pili signaling differs in other species. In *Caulobacter crescentus*, the response to mechanical force generally happens through the combined action of the pilis and the flagellar motor and activates the production of a sticky polar holdfast to promote irreversible surface attachment.^34,35^ In this case, intracellular c-di-GMP levels are increased by diguanylate cyclase (DgcB), which activates holdfast biogenesis. In *B. subtilis*, surface attachment via T4P activates the two-component system DegS–DegU independently of second messengers to promote biofilm production.^36^ In this case, the histidine kinase Deg S phosphorylates DegU (DegU-P) to initiate the transcription of the genes encoding poly-γ-dl-glutamic acid, a biofilm matrix component.

A growing number of studies indicate a significant role for the bacteria flagella in surface sensing and mechanotransduction. For example, in *P. aeruginosa*, the flagellum stator motor proteins MotA and MotB initiate the response to surface contact by inducing c-di-GMP, which then stimulates T4P and adhesion.^37^ Multiple types of bacteria can express two distinct flagella systems: the polar flagellum required for swimming motility and separate lateral flagella induced by viscous media or surfaces that facilitate swarming.^38^ The polar flagellum differs in subunit composition from the lateral flagellar system. The pathogen *Vibrio parahaemolyticus* controls the gene expression of a single polar flagellum and multiple lateral flagella, which depends on the substrate and the nutrient availability of iron. Interestingly, the deep-sea bacteria *Photobacterium profundum* responds to changes in pressure by modulating the relative expression of genes encoding polar and lateral flagella: at low pressure, *P. profundum* uses polar flagella to swim, whereas at high pressure, it activates the synthesis of lateral flagella needed to move at high pressures.^39^ The deep sea bacterium *Shewanella piezotolerans* can also sense and respond to changes in pressure, transitioning to a swarming phenotype at high pressure.^40^

Another mechanism by which bacteria may sense surface adhesion is the monitoring cell envelope proteins.^41,42^ In *E. coli*, the Cpx signaling system helps maintain the cell envelope integrity by sensing misfolded proteins and activating gene transcription for factors that repair the damage. The Cpx system is a two-component system. CpxA is a membrane-spanning histidine kinase that transmits information about the membrane status to its cytoplasmic response regulator, CpxR, to induce the transcription of genes.^42^ The Cpx system is sensitive to external perturbations of the cell envelope and physical changes that occur during adhesion of the cell to the substrate. For example, CpxA is activated when *E. coli* binds to hydrophobic surfaces, and this activation subsequently upregulates the outer-membrane lipoprotein NlpE.^41^

Identifying how bacteria readout surface cues is critical to interpreting the role of physical stimuli in pathogenic bacteria and microbial infections of host cells and tissues. Enterohemorrhagic *E. coli* (EHEC) is a common intestinal pathogen that causes severe intestinal infections.^43^ Upon host cell contact and flows in the intestine, the EHEC strain O157:H7 upregulates expression of the ler1 gene, genes of Lrha-dependent pathway, and type III secretion components.^44^ In another study, fluid flow was also shown to increase ler1 expression in an EHEC strain.^45^ Uropathogenic *E. coli* (UPEC), the main cause of urinary tract infections, upregulates the rpoH gene to induce EPS production and promote biofilm growth at the uroepithelium.^46^ Another pathogen, *Vibrio cholerae*, which causes a deadly diarrheal illness, regulates its biofilm formation by mediating the expression of matrix protein Rbm A, known for tuning the matrix mechanics and consequently multicellular accumulation within biofilms.^47^ *V. cholerae* also produces the other biofilm matrix proteins Bap1 and RbmC, which helps adhere the biofilm to external surfaces.^48^

## IV. MECHANICAL AND MORPHOLOGICAL METHODS

To shed light on how cells respond to physical forces, it is important to measure the mechanical properties of the cells and the forces they generate. There are several experimental methods used to determine the mechanical properties of living materials and how they respond to mechanical cues. These experimental methods can be broadly characterized as either top-down, in which external perturbations are applied to the system, or bottom-up, non-invasively analyzing cell behavior in a local environment, which when combined with microscopy imaging methods can differentiate between molecular mechanisms of bacteria force generation.

### A. Top-down mechanical assays

#### 1. Bulk rheology

**FIG. 5.**
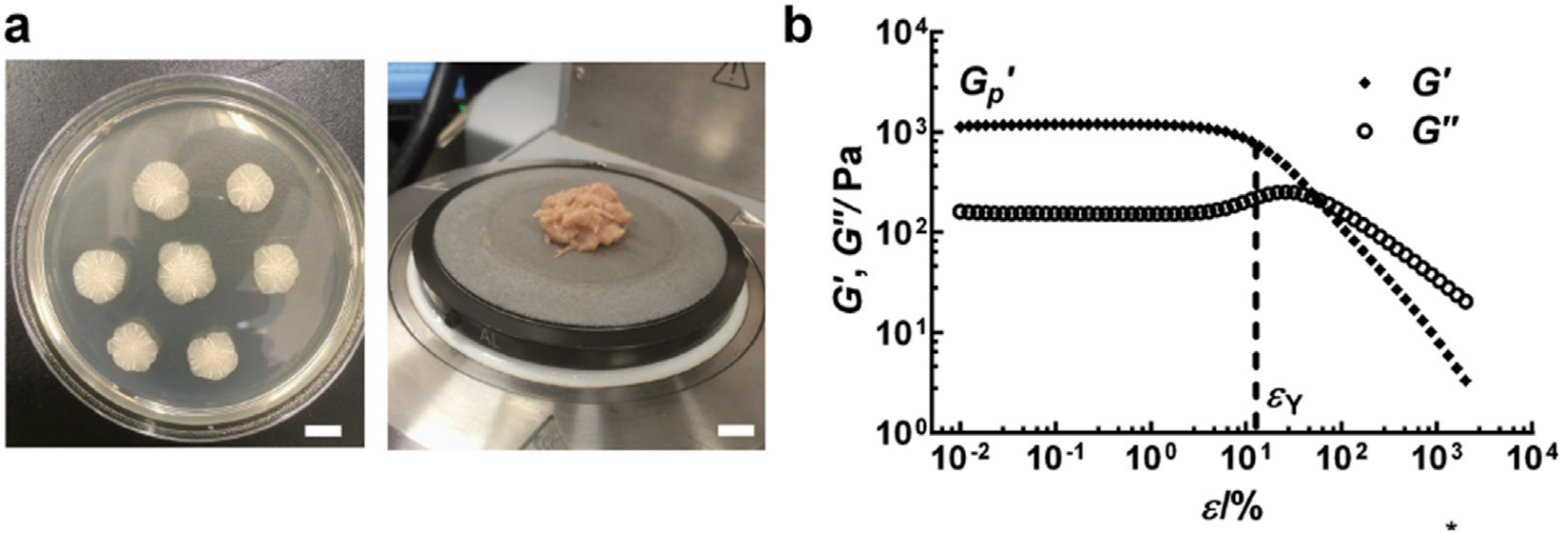
Bulk rheology of biofilms. (a) Bulk rheology of biofilms can be performed by growing up many bacteria colonies and gathering them as one sample on the rheometer plate, as shown for *V. cholerae* biofilms (b) Biofilms exhibit viscoelastic behavior, characterized by a shear storage modulus G′ and a viscous loss modulus G″. For low shear strains, the biofilm has a nearly constant shear modulus (∼1000 Pa), which begins to decrease above a critical shear strain value. This is referred to as a yielding point, above which more strain makes the sample softer and more viscous-like. Figure adopted from Ref. 50. Reproduced with permission from Yan *et al.*, Adv. Mater. **30**, 1804153 (2018). Copyright 2018 Authors, licensed under a Creative Commons Attribution (CC BY) license.

Rheology is the study of flow and deformation of materials, and a rheometer is a tool that applies external stresses or strains on a sample boundary to measure its mechanical response. Biofilms have complex viscoelastic mechanical properties, exhibiting both solid-like and fluid-like characteristics. Oscillatory rheology quantifies viscoelastic properties through two central metrics: a storage modulus G' that characterizes the solid-like behavior and a loss modulus G″ that characterizes the fluid-like behavior. A rheometer performs a bulk measurement, averaging over relatively large samples (100 *μ*l+ per sample). Rheological studies of biofilms or isolated EPS, thus, often require growing up large numbers of biofilms on many agar plates and transferring them together into the rheometer (Fig. 5).^26,49,50^

#### 2. Microrheology

In contrast to bulk rheology, microrheology examines local mechanical properties of small samples. In microrheology, the sample is embedded with small probe particles (spheres) of glass, steel, or polystyrene, typically 0.1–10 *μ*m in diameter. Due to the small size of the probe particles, they exhibit random Bownian fluctuations. The Brownian diffusion of the particles depends on the local viscosity and is resisted by any elastic component of the microenvironment, allowing one to compute the viscous and elastic modulus of a sample via passive microrheology techniques.^51^ In active microrheology, magnetic or optical tweezers apply external forces to the probe particles. By measuring displacements of the trapped particle resulting from the applied forces, the elastic G′ and loss moduli G″ of the sample can be computed.^52,53^

Studies using microrheology techniques have generated many new insights into the local microscopic environment of bacterial biofilms. An early study examined *P. aeruginosa* and *S. aureus* biofilms and found they exhibited power-law rheology, consistent with other dense colloidal suspensions and soft glassy materials.^54^ Furthermore, the biofilms were rheologically inhomogeneous on the micrometer scale, due to initial adhesion and arrangement of individual bacteria and the development of large irregular cell clusters. Interestingly, *S. aureus* biofilms become less compliant during growth, and more compliant during starvation. Microrheology can also be used to glean the mechanical contribution of individual components of the EPS matrix. A study by Chew *et al.* using microrheology and genetic approaches showed that the major exopolysaccharide Psl in *P. aeruginosa* biofilms increased biofilm elasticity and effective cross-linking in the matrix, whereas the exopolysaccharide Pel reduced effective cross-linking within the matrix.^55^ As biofilms mature and grow into large structures, they must solve a new problem: the transport of nutrients throughout the entirety of the biofilm. Microparticle tracking revealed that *E. coli* biofilms contain micrometer-scale, fluid-filled channels that penetrate throughout the biofilm, permeabilizing it and enabling the transport of biological material.^56^

#### 3. Atomic force microscopy

The first step of biofilm formation involves the initial attachment of individual bacteria to a surface. Understanding the molecular mechanics of coupling bacteria to a surface is, therefore, critical to understanding how biofilms begin. Scanning force microscopy, and in particular, atomic force microscopy (AFM), has been a popular and powerful tool for these studies. In studies involving AFM, a cantilever tip is used as a scanning probe to measure the force of interaction between the cantilever tip and the sample (e.g., individual cell surface or bulk biofilm). Deflection of the cantilever can be used to obtain simple stress, strain, and moduli with appropriate contact models.^57,58^

**FIG. 6.**
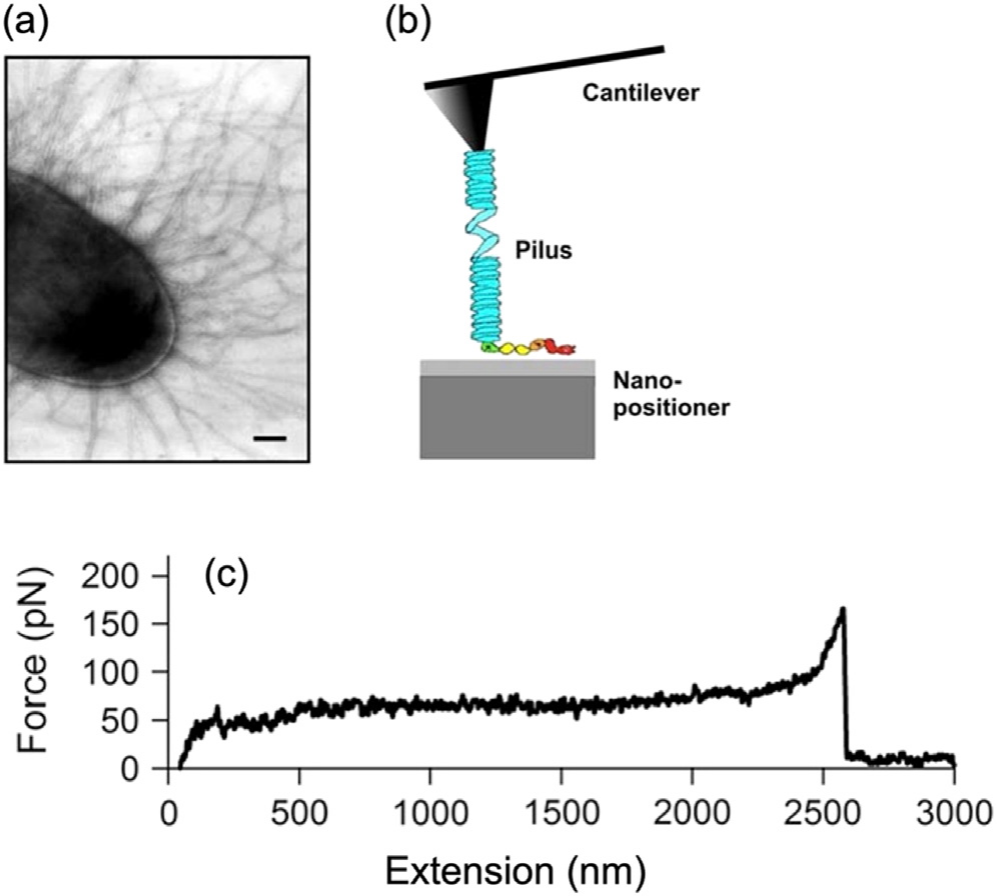
AFM characterization of bacteria appendages. (a) Electron micrograph image of type I pili in uropathogenic *E. coli*. (b) Schematic of experimental setup with AFM cantilever tip gripping a type 1 pili filament. (c) Force-extension curve showing a force plateau that corresponds to unwinding of the pilus sub-units at a constant force. Figure adopted from Ref. 61. Reproduced with permission from Miller *et al.*, Biophys. J. **91**, 3848 (2006). Copyright 2006 Authors, licensed under a Creative Commons Attribution (CC BY) license.

Specific physicochemical forces and biological factors can be investigated by coating the AFM tip with proteins of interest or examining the force between a coated surface and a bacteria cell fixed to the tip of the cantilever.^59,60^ The effectiveness of the AFM technique has been exemplified in studies such as Miller *et al.*^61^ and Forero *et al.* in which the force-extension curve of specific cellular adhesive appendages was measured and related to their molecular structure (Fig. 6).^62^ Miller *et al.* found that type 1 pili of uropathogenic *E. coli* readily extend under applies forces of 60 pN, consistent with unwinding of the pilus rod's helical quaternary structure.^61^ Similarly, Forero *et al.* found that fimbriae rapidly elongate at applied forces of approximately 60 pN and above, consistent with uncoiling of the fimbriae's helical structure.^62^ Taken together, these works highlight how bacterial appendages can absorb physiologically relevant forces, such as shear flows that they would encounter when adhered to surfaces.

#### 4. Flow assays

Fluid flow is a ubiquitous feature of most bacteria environments and can be a source of new nutrients for surface-attached colonies. External fluid flows impact both the adhesion rates of individual cells on a surface and the resulting bulk biofilm shape. Several flow assays have been developed to study biofilm formation, such as shear flow cells, microfluidic channels, and bioreactors, which provide cells with a continuous stream of nutrients. Interestingly, multiple types of bacteria adhere more rapidly and more strongly to channel walls when subject to stronger shear flows.^63–65^ In *E. coli*, this effect has been attributed to the fimbrial adhesin FimH, which is capable of the conformational changes that enhance its bond strength with increasing tensile mechanical forces (e.g., a catch bond) due to shear flows.^63^ Once bacteria begin dividing and forming colonies, shear flow can dramatically alter the shape of the emergent biofilm structure. While some biofilms grown in a low-shear environment form approximately symmetrical hemispheres, at higher shear the biofilms form more elongated droplet-like shapes, which align with the direction of the shear flow.^66^ Over time, biofilms can form filamentous streamers particularly around surface corners or structures^12,28,67^ and micro-colonies that can detach and roll along the surface with the flow.^68^ Interestingly, recent studies using microfluidic flow assays and transcriptomic techniques have discovered a novel operon, named flow-regulated operon (fro), which is rapidly upregulated in response to flow in *P. aeruginosa.*^69^ These studies showed fro-dependent flow sensing is a kinematic process and did not depend on any know surface adhesion proteins.

### B. Bottom-up mechanical assays

#### 1. Varying substrate mechanical properties

Bacteria can adhere to and colonize a variety of surface types as rigid as glass or medical implants and as flexible as agar and soft tissues. Most biofilm experiments in the lab use semi-solid agar substrates to culture bacteria. Agar, a gelatin hydrogel isolated from marine algae, was introduced in 1882 by Angelina Fanny Hesse and Robert Koch and gained popularity because it is inert to bacterial degradation.^70^ A common feature of biofilm growth in the laboratory is that they are more spread on soft agar substrates compared to stiff agar substrates.^71–73^ On stiff agar, the pore size is smaller and the rate of nutrient transport through the substrate and to the biofilm decreases. A number of studies have attributed this inhibited biofilm growth on stiff agar to lack of nutrients than a direct effect of the stiffness.^72,74^ While agar is overwhelmingly used for biofilm studies, its use for bacteria mechanosensing studies is problematic, because it is difficult to systemically control and manipulate physical features of the agar in a systemic way because substrate stiffness, network pore size, and substrate viscoelasticity are all coupled together with agar concentration. This means that the isolated effects of physical features, such as substrate stiffness for example, cannot be isolated as a separate experimental property and studied in a way that can directly and unambiguously relate it to the bacterial response.

**FIG. 7.**
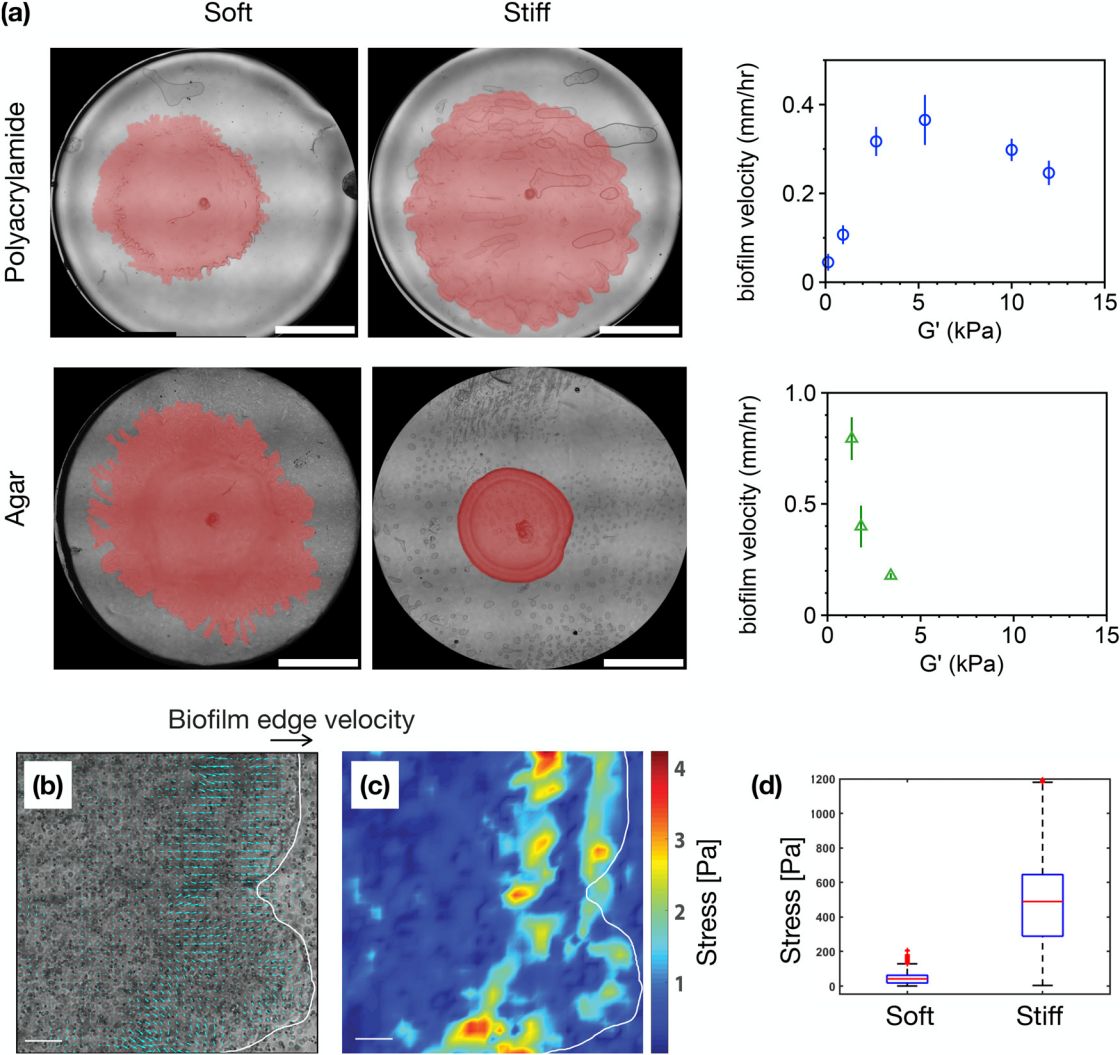
Effect of substrate stiffness on bacteria colony expansion. (a) Characterization of *Serratia marcescens* colony expansion on soft and stiff agar substrates vs soft and stiff synthetic polyacrylamide (PAA) substrates. While colony growth is slower on denser, stiffer agar, colony expansion increases with increasing stiffness on linearly elastic PAA gels. (b) Displacement of the substrates are monitored via fiducial markers, allowing computation of a corresponding (c) stress map. (d) The stress generated by the bacteria colony is greater on stiff PAA substrates (G′ = 5 kPa) compared to soft ones (G′ = 0.5 kPa). Figure adopted from Ref. 76. Reproduced with permission from Asp *et al.*, PNAS Nexus, **1**, pgac025 (2022). Copyright 2022 Authors, licensed under a Creative Commons Attribution (CC BY) license.

Motivated to identify the mechanisms by which bacteria sense and respond to physical properties of their environment, new studies are emerging using synthetic hydrogel substrates, such as polyacrylamide (PAA),^75,76^ polyethylene glycol (PEG),^77,78^ or polydimethylsiloxane (PDMS),^79^ with more discrete and tunable mechanical and chemical properties on which to culture bacteria. These hydrogel substrates are revealing intricate (and sometimes contradictory) ways in which the microenvironment properties affect bacteria attachment and biofilm growth. For example, increasing substrate stiffness has been found to either enhance^78^ or hinder^80^ bacteria attachment to surfaces. Interestingly, at the collective colony level, recent studies from our group using linear elastic PAA gels showed that bacteria colonies spread out faster on stiffer substrates compared to softer ones, which is the opposite of the results on conventional agar substrates (Fig. 7).^76^ These works highlight the importance of physical cues in the bacteria environment in bacteria behavior, which can result in a wide variety of bacteria outcomes.

#### 2. Traction force microscopy (TFM) and nanopillars

Recent work has led to the discovery that bacteria, particularly collective groups of bacteria, can generate sufficient forces to deform soft substrates on which they grow.^76,77,81^ Traction force microscopy (TFM) is a technique originally developed to study forces generated by animal cells^82^ that within the last few years has begun being adapted to bacteria systems (Fig. 7). TFM uses embedded fluorescent tracers (0.01–10 *μ*m beads) to track the displacements of the substrate. Polyacrylamide gels (or other linearly elastic hydrogels) are widely used as substrates for TFM,^83^ because their well-defined elastic properties allow the displacement fields to be related to the cell applied stresses through continuum elastic theory.^84^

One of the first TFM studies with bacteria used *M. xanthus*. Sabass *et al.* found that twitching bacterial groups produced traction hotspots of approximately 100 pN, twice as large as forces from individual twitching cells.^81^ In subsequent studies, Duvernoy *et al.* showed that growing colonies of *E. coli* and *P. aeruginosa* generate heterogenous and dynamic adhesive hot spots of approximately 200 Pa.^85^ Biofilm-forming colonies, reinforced by the EPS matrix, are able to generate much larger stresses, up to 100 kPa.^77^ We recently investigated whether bacteria force-generation depended on the mechanical properties of their underlying substrate. We found that *Serratia marcesens* colonies generated 10-fold higher stress when grown on stiff substrates compared to soft ones,^76^ highlighting the importance of the substrate in the ability of bacteria to produce force. Like TFM, micro-posts or nanopillars, which can be pulled on and deflected by cells, can be used to measure cell-generated forces. In a study by Sahoo *et al.* for instance, asymmetric bacterial forces were mapped around individual cells using nanowire arrays.^86^ The largest adhesion forces (50 nN) were found at the cell poles and were reinforced by EPS filaments. Altogether, while the bacteria force machinery is still far from completely described, collectively TFM and pillar studies are beginning to delineate the basic length, force, and time scales relevant to force-generation and surface sensing, which are necessary to distinguish between different molecular mechanisms.

#### 3. Topographic patterning and surface roughness

Recent advances in surface engineering have enabled studies on the effect of topographic patterns and surface roughness on bacteria and biofilm development. In general, surface roughness tends to increase bacteria adhesion and biofilm formation, adding surface area for cells to attach to and providing protection against shear forces.^87–89^ Topological surface features, however, can be tuned to either favor or hinder bacterial adhesions. For instance, Perni *et al.* using cone-shaped patterns on silicone surfaces showed *E. coli* and *S. epidermidis* showed bacteria predominantly localized in cone valleys but not on cone tops.^90^ In addition to surface adhesion, changes in surface topography can lead to morphological and genetic changes in the cell. Rizzeo *et al.* found that *E. coli* typically lost their type-1 fimbriae filaments on nanostructured substrates compared to *E. coli* on flat rigid surfaces, such as glass.^91^ Furthermore, *E. coli* on nanorough substrates triggered an increase in expression of proteins involved in stress processes and defense mechanisms.^91^ Another significant aspect of surface topology is that it can changeover time. Dynamic changes in surface topology induced by mechanical buckling of PDMS^92^ or shape-memory polymer techniques^93^ have been shown to significantly inhibit biofilm build-up in *P. aeruginosa*, *E. coli*, and *S. aureus*.

### C. Imaging

Gaining a deeper understanding of bacterial mechanics will require advanced imaging techniques. Imaging bacteria can be challenging, as individual cells are small (cell volumes ∼0.4–3 *μ*m^3^^94^) and often dynamic (swimming speeds ∼10–100 *μ*m/s). Due to their thin membrane, bacteria are transparent with light microscopy;^95^ thus, phase contrast and dark field microscopy are more often used to image cells for better contrast. Dark field microscopy has long been used to image swimming bacteria, as it can resolve individual unstained flagella in swimming cells.^96^

Labeling a protein with a fluorescent marker allows for single-molecule tracking or spatiotemporal visualization of the expression of a gene of interest. Single-molecule tracking experiments have helped elucidate the role of FtsZ in the mechanics of cell division^97^ and have identified that mechanical stress on the cell envelope can lead to increased metal toxicity by causing disassembly of the CusCBA efflux system in *E. coli.*^98^ Similarly, reporter genes such as beta-galactosidase or green fluorescent protein (GFP) can be used to assess promoter activity in the regulatory region of a gene of interest. For example, reporter genes have been used to demonstrate the role of the P_R_ and P_H_ promoters in enterococcal resistance to glycopeptide presence in the environment.^99^ Fluorescence microscopy techniques such as fluorescence recovery after photobleaching (FRAP) can be used to track the assembly and disassembly of proteins as well. In a recent study by Koch *et al.* for instance, FRAP was used to observe the dynamics of PilA, a major subunit of pili, and showed that the slow diffusion of PilA leads to concentration changes at the base of T4P that change with substrate stiffness.^100^

Scanning and transmission electron microscopy (SEM and TEM, respectively) are progressively being recognized as powerful tools to understand organelle ultrastructure. Both techniques use electrons as an excitation source, which can probe length scales on the order of nanometers, smaller than what is resolvable with visible light. These techniques have provided a more detailed characterization of biofilm form and structure,^101,102^ cell wall, and membrane damages upon treatments with anti-bacterial agents,^103^ and extracellular DNA content.^104^

White light interferometry is a content- and label-free method of mapping biofilm surfaces. The height profiles and surface roughness of biofilms are tied to local cell death and reproduction, which can be difficult to measure directly.^105,106^ Mapping the 3D structure of biofilms is an important part of understanding the mechanical relationship between the biofilms and its environment and modeling biofilm growth and development.

## V. Methods for characterizing changes in gene expression and protein activity in response to physical features of the microenvironment

It is well documented that bacteria can respond to changes in the chemical environment by up- or down-regulating the expression of relevant genes. A classic example is the *lac* operon, which upregulates the expression of the genes required for lactose metabolism when the concentrations of lactose and glucose in the environment are high and low, respectively.^107,108^ In response to variations in temperature, cells increase expression of heat shock proteins and molecular chaperones that ensure proper protein folding.^109,110^ It follows that bacteria would also respond to mechanical forces in the environment by differentially regulating gene expression. Research indicates that substrate properties can significantly affect transcriptional profiling. For instance, 10% of all genes in the *S. enterica* genome are differentially regulated between growth on soft and stiff agar.^111^

For some bacterial responses to the environment, there may be hypotheses about what genes are involved. For example, if different environments cause a noticeable increase in colony expansion, genes regulating motility machinery may serve as promising candidates for further study. If potential candidate genes can be identified based on their predicted function from previous work or their homology to other well-characterized genes, targeted approaches can be taken to confirm whether a gene plays a role in responding to physical features of the microenvironment. RT-qPCR is a molecular technique for quantifying the amount of transcript of a particular gene and can be used to assess differential expression of a gene temporally and under differing environmental conditions, indicating a gene's potential involvement in a process or behavior of interest.^112^ This technique has identified genes involved in abiotic surface attachment in *Listeria monocytogenes,*^113^ and those that are differentially regulated during attachment to surfaces with different physical properties.^114^ Once specific genes of interest are identified, they can be spatiotemporally tracked by generating cell lines with fluorescent gene reporters. Additionally, knockout or knockdown mutant strains can help to elucidate the role of genes in the bacterial response to a particular cue or stress. If a knockout or knockdown mutant strain loses its ability to respond to an aspect of the physical environment in a similar manner as is seen in the wild-type strain, the mutated gene is more likely to play a role in that response.

In the absence of any identified “candidate” genes, or in the interest of a more comprehensive approach to probe the global dynamics of expression in response to physical features of the environment, transcriptomics can be used to detect changes in all expressed genes. Bacterial transcriptomics typically employs the technique called RNAseq, which involves the extraction of total RNA from samples under specific conditions, reverse transcription of transcripts into cDNA for sequencing, and mapping sequenced reads to a reference genome to determine the abundance of transcripts at each region of the genome.^115^ RNAseq has already been used to capture changes in gene expression in response to the physical environment, such as during the transition from planktonic to a surface-attached lifestyle,^116^ in the harsh landscape of the human host,^117^ under antibiotic treatment,^118,119^ in minimal oxygen,^120^ and under many other conditions.

A second approach can be taken using quantitative proteomics, which measures total protein abundance in different samples through total protein extraction and identification and quantification of proteins using mass spectrometry.^121^ Bacterial proteomics has previously been used to identify proteins involved in increased virulence and in response to the presence of antibiotics.^122^ A third and more traditional approach, the mutagenesis screen, remains an important experimental tool to identify genes involved in response to the physical environment. Random mutagenesis, induced with mutagens like UV light or transposons, produces many mutant strains that can be selected or screened for phenotypic differences, potentially revealing genes important for response to the physical environment.

The power of these genetic and molecular techniques to associate differential gene expression or protein abundance with a particular response to the environment has strong implications for the genotype-to-phenotype problem by assigning functions to genes and improving understanding of the interaction between the genome and the environment. Techniques, such as targeted mutagenesis, RT-qPCR, and the use of reporter genes, can be used to investigate the role of candidate genes in the biological response to different environmental conditions. Transcriptomics, proteomics, and genetic screening can provide a more comprehensive picture of the ways in which the physical environment changes gene expression or protein abundance and can implicate genes of previously unknown function in biological processes of interest, highlighting candidates for future work.

## VI. CHALLENGES AND FUTURE PERSPECTIVES

### A. Model reduction as a tool for taming complexity in mechanobiological models

As methods improve to screen large sets of genes and assess their phenotypic impact, analysis of larger and larger datasets becomes essential. Likewise, as more hypothetical mechanisms for the cascading impacts of genetic effects are identified, the quantitative models that capture these mechanisms become more complex and more difficult to interpret.

In some cases, known phenotypic categories may not be exhaustive, or none may be present *a priori*, necessitating analytical methods that robustly simplify high-dimensional descriptions of behavior. A powerful technique of dimensional reduction that requires no input parameters is Principal Component Analysis (PCA). This tool identifies among all the directions in the high-dimensional space just those directions that have the most variance and are, thus, the most relevant to cause-and-effect investigation. This can also have the effect of revealing correlations or clustering in multivariate data, important structural properties of a dataset, that are often not immediately clear.^123^ Because of its generality, low computational cost, and transparent interpretation, PCA is being used to quantify phenotype in bacterial development,^124^ rank phenotypic variance in cell morphology,^125^ and assess the physical impact of antibiotics in high-throughput assays,^126^ among other applications.

Another potentially useful approach is machine learning. This is a family of techniques that excel at automating subtle and complex functions when they are given sufficiently large sets of input and output data to learn from, called “training data.” This learned function can then be applied to new input data that the machine learning algorithm was not trained on.^127^ For example, when given many images of cellular colonies and the positions of cells in those images, a machine learning algorithm such as a Support Vector Machine (SVM) can learn to segment new colony images automatically.^128–130^ Similarly, if genetic inputs and phenotypic outputs are known across a large training dataset, machine learning can be used to predict which phenotypic category may result from a given genotype even when the phenotype is not yet known. This prediction is independent of any biological model and depends on a finite number of distinct, expected phenotypes. Although so-called “unsupervised” machine learning can be used to automatically identify patterns in a dataset,^131^ these categories may not be clearly associated with any phenotype. A trade-off with machine learning techniques is the accuracy of the predictive model vs clarity of how categorization is achieved. Refinements of machine learning, such as Classification and Regression Trees (CART) or Set Covering Machines (SCM) produce explicitly rules-based models designed to have a clear interpretation, at a slight cost of accuracy. One study on antibiotic resistance in bacteria used a public database of bacterial genomes that were tagged with antibiotic resistance to produce many rules-based models across 12 species. For instance, *M. tuberculosis* resistance to kanamycin was predicted with 93.7% accuracy over a dataset of 5000 genomes.^132^ Machine learning has also been applied in other genome wide association studies, such as the identification of genes associated with bacterial virulence.^133^

Beyond datasets, models that test mechanobiological mechanisms have high complexity in need of interpretation. When genetic information is included in physical models of living systems, many new parameters are introduced, such as rate constants of production for relevant signaling proteins. Often these parameters are not practical to measure directly, and many may have little to no impact on an emergent phenotype. Model reduction techniques from the study of so-called “sloppy models” are an attractive way forward in such cases. By choosing a target output, or phenotype, the myriad genetic and environmental inputs can be reduced to uncover only those combinations of input parameters that strongly affect the resulting phenotype.^134^ Techniques such as the manifold boundary approximation method have been successfully used to reduce cell signaling and ion channel models.^135^ With key parameters and simplified signaling networks identified, mechanobiological studies into the genotype-to-phenotype problem can be made more predictive and transparent.

### B. Disease relevance

Infection of human cells by bacteria pathogens typically proceeds by bacteria binding to a specific cell surface receptor protein. The chemical reactions between bacteria and host cell receptors are, thus, generally assumed to dominate host cell invasion. However, several studies are now beginning to reveal that microbial infections only proceed when certain mechanical conditions are met, highlighting the importance of mechanical interactions between host cells and pathogenic bacteria.

**FIG. 8.**
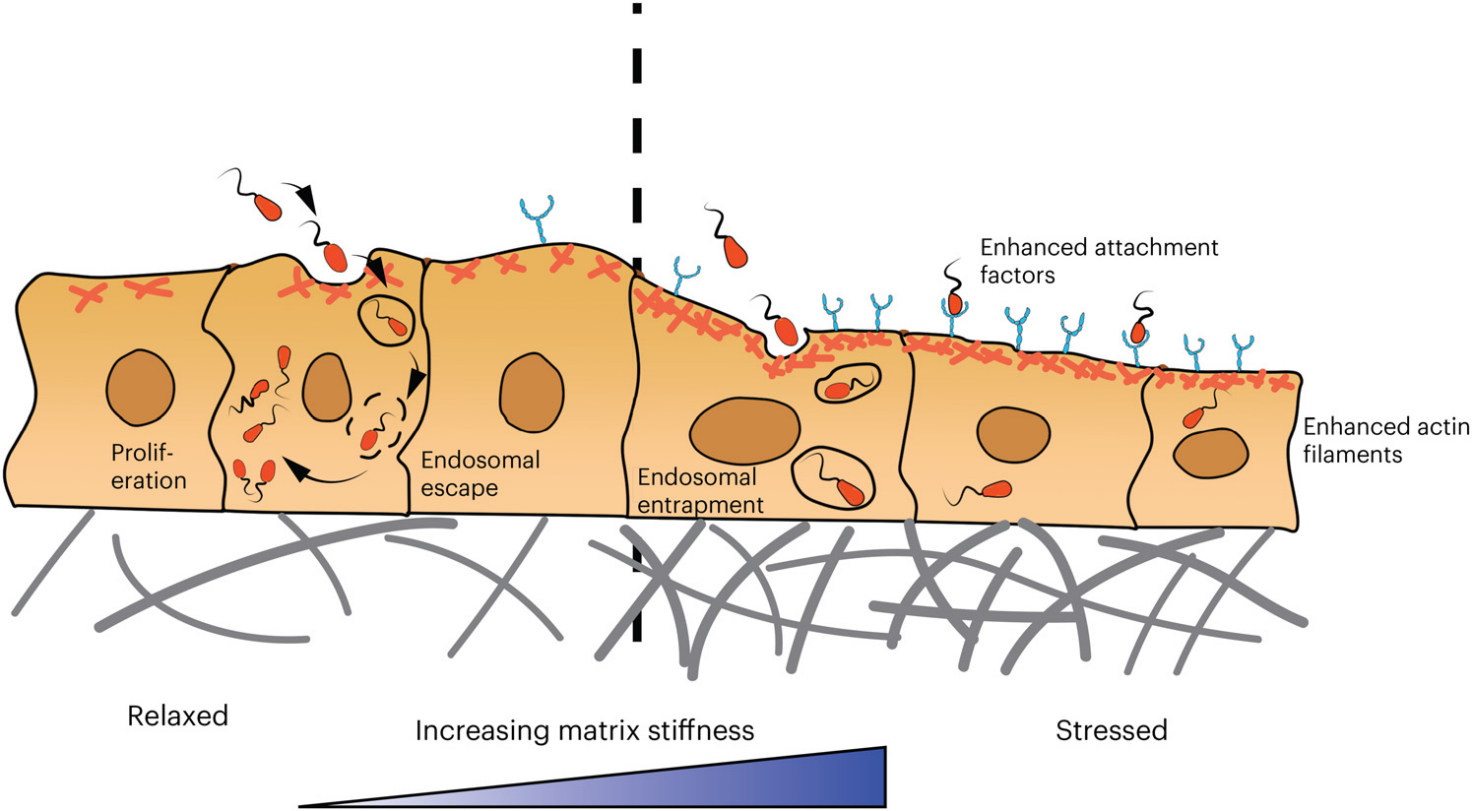
Effects of substrate stiffness on host cell invasion. Multiple studies have shown that increased matrix stiffness increases the number of host cells infected by bacteria pathogens.^138,140^ Host cells change their morphology and behavior on stiff substrates, in many aspects making them more susceptible to bacteria invasion. For instance, when cultured on stiff substrates compared to soft ones, endothelial cells express more extracellular attachment factors, such as extracellular vimentin, that bind bacteria and facilitate host cell uptake.^140^ Further, the host cell actin cytoskeleton—which is more prominent on stiff substrates—colocalizes at sites of bacteria invasion.^138^ While more host cells are infected on stiff substrates, the number of bacteria in infected cells can actually be higher on soft substrates.^138^ This may be due in part to endosomal escape, which is significantly more prevalent for host cells cultured on physiologically soft substrates compared to rigid glass or tissue culture plastic.^141^

Most of the work in this field has been done *in vitro* by studying the interactions between bacteria and an external substrate directly or bacteria and host cells attached to substrates. The effects of the tissue microenvironment on bacterial infections can generally be classified into two possible categories. The first is by their effects on the bacteria themselves, and the second on the behavior of the host cells (and, thus, their susceptibility to bacteria) (Fig. 8). From the perspective of pathogenic bacteria, we have already described in this article how substrate stiffness and roughness can impact bacteria adhesion,^78,80,88,90^ motility,^71,72,76^ and differentiation to a virulent biofilm state.^32,136^ Recent studies have also gathered evidence that environmental stiffness can impact other factors relevant to bacterial disease. For instance, bacteria cultured on stiff substrates are more effectively removed by macrophages via phagocytosis^137^ and are less susceptible to antibiotic treatments,^138^ as compared to bacteria grown on soft substrates. In a recent study by *Pierrat *et al.**, the physical interactions between *E. coli* and human cells were examined.^139^ In this study, the host cell's glycocalyx was shown to act as a physical shield, blocking bacteria from the host cell membrane. From these studies, it is tempting to posit that some of these physical interactions impact bacteria infection rates *in vivo*.

From the perspective of host cells, multiple studies now indicate that increased host matrix stiffness enhances bacteria uptake.^138,140^ For instance, a study by Bastounis *et al.* showed that *L. monocytogenes* can infect human endothelial cells better when the endothelial cells are cultured on stiffer substrates compared to softer ones.^140^ In this study, extracellular vimentin on the cell surface was identified as an attachment factor for *L. monocytogenes* to bind and facilitate uptake in a substrate-stiffness dependent manner.^140^ Liu *et al.* also found that the number of human cells infected by different bacteria increased with increasing substrate stiffness.^138^ Here, bacteria invasion was correlated with the host cell's actin cytoskeleton: bacteria co-localized in regions of F-actin and high cell stress, which were more abundant on stiff substrates than soft substrates. Matrix stiffness has also been shown to regulate other host cell factors relevant to infection, such as the activity of macrophages^137^ and the ability of antibiotics to accumulate inside the host cell to target bacteria.^138^

An illustrative example of the effects of tissue stiffness on bacteria infection comes from the study of Moorthy *et al.* on endosomal escape of bacteria within host cells.^141^ *In vivo*, UPEC is known to be taken up into host cells by endocytosis followed by endosomal escape and proliferation in the cell cytoplasm. For many years, this important feature of infection could not be readily observed *in vitro*: infection of epithelial cells grown on tissue culture plastic or glass led to UPEC trapped within their entering endosomal vesicles, where proliferation is limited. Moorthy *et al.* demonstrated that by culturing epithelial cells on soft hydrogel substrates, a drastic increase in UPEC endosomal escape and proliferation within cells could be observed.

Overall, these studies highlight the significance of studying cells under the physiologically soft conditions that mimic the *in vitro* environment. The field of mechanobiology provides new avenues of research for investigating host cell-microbe interactions, which could be crucial to revealing new mechanistic insights of infection and identifying new targets for therapy.

### C. Mechanosynthetic biology

The combined use of -omics methods and mutagenesis screens has proven very effective at generating lists of candidate genes and building maps that display interactions at systems-scale. These maps can be large and complicated enough to represent entire signal transduction pathways, including mechanotransduction pathways. Manifesting a subdiscipline like mechanosystems biology suffers from the same impediments as all systems biology; an organism's inherent complexity, plasticity, and non-linearity are typically not represented in experimental data and, therefore, cannot be part of interaction maps. Static, non-bifurcating, linear approximations of signal transduction pathways are very important for understanding developmental biology, but they typically do not scale-up to whole genomes and provide little of the predictive accuracy required to make a nascent engineering discipline like mechanosynthetic biology feasible. Nevertheless, the potential power of mechanosynthetic biology makes enabling such a discipline a worthy goal. The idea that we could “design” a microbial genome, which would produce an organism that interacted with the physical environment in specific predetermined ways has enormous applications for almost every field, including medicine, agriculture, and industry.

## VII. CONCLUSIONS

Many in the life science community are now beginning to recognize that physical features of a biological system can have as great an effect on its behavior as its composition of genes and proteins. Bacteria sense not only biochemical signals but physical cues, such as force, flow, and surface stiffness. These physical cues are translated into biochemical signals by mechanosensitive organelles and protein complexes that interact directly with the cell's “outside world,” allowing cells to adapt to physical cues by modulating their adhesion, motility, and changing their morphological features. These processes underly the ability of bacteria to colonize diverse environments and are central to defining the physiology of individual bacteria cells and collective bacteria colonies. Mechanobiology has many quantitative techniques for characterizing forces that can be used to identify the molecular and biophysical mechanisms by which bacteria sense and respond to their environment. This is an interdisciplinary problem that will require teams of biologists, physicists, chemists, engineers, and mathematical and computational modelers working together to decipher and advance our knowledge of how cellular phenotypes emerge from cell genomes and environmental conditions.

## Data Availability

Data sharing is not applicable to this article as no new data were created or analyzed in this study.

## References

[1] A. Xavier da Silveira Dos Santos and P. Liberali , “ From single cells to tissue self-organization,” FEBS J. 286, 1495–1513 (2019). 10.1111/febs.14694 30390414 PMC6519261

[2] P. A. Janmey and R. T. Miller , “ Mechanisms of mechanical signaling in development and disease,” J. Cell Sci. 124, 9–18 (2011). 10.1242/jcs.071001 21172819 PMC3001405

[3] W. Zheng and J. R. Holt , “ The mechanosensory transduction machinery in inner ear hair cells,” Annu. Rev. Biophys. 50, 31–51 (2021). 10.1146/annurev-biophys-062420-081842 33285080 PMC8163026

[4] B. Martinac , M. Buechner , A. H. Delcour , J. Adler , and C. Kung , “ Pressure-sensitive ion channel in *Escherichia coli*,” Proc. Natl. Acad. Sci. U. S. A. 84, 2297–2301 (1987). 10.1073/pnas.84.8.2297 2436228 PMC304637

[5] K. R. Levental , H. Yu , L. Kass , J. N. Lakins , M. Egeblad , J. T. Erler , S. F. T. Fong , K. Csiszar , A. Giaccia , W. Weninger *et al.*, “ Matrix crosslinking forces tumor progression by enhancing integrin signaling,” Cell 139, 891–906 (2009). 10.1016/j.cell.2009.10.027 19931152 PMC2788004

[6] D. B. Kearns , “ A field guide to bacterial swarming motility,” Nat. Rev. Microbiol. 8, 634–644 (2010). 10.1038/nrmicro2405 20694026 PMC3135019

[7] A. E. Patteson , A. Gopinath , and P. E. Arratia , “ The propagation of active-passive interfaces in bacterial swarms,” Nat. Commun. 9(1), 5373 (2018). 10.1038/s41467-018-07781-y 30560867 PMC6299137

[8] N. C. Darnton , L. Turner , S. Rojevsky , and H. C. Berg , “ Dynamics of bacterial swarming,” Biophys. J. 98, 2082–2090 (2010). 10.1016/j.bpj.2010.01.053 20483315 PMC2872219

[9] J. Pérez , A. Moraleda‐Muñoz , F. J. Marcos‐Torres , and J. Muñoz‐Dorado , “ Bacterial predation: 75 years and counting!,” Environ. Microbiol. 18, 766–779 (2016). 10.1111/1462-2920.13171 26663201

[10] S. Thiery and C. Kaimer , “ The predation strategy of *Myxococcus xanthus*,” Front. Microbiol. 11(2), 1–7 (2020). 32010119 10.3389/fmicb.2020.00002 PMC6971385

[11] G. Liu , A. Patch , F. Bahar , D. Yllanes , R. D. Welch , M. C. Marchetti , S. Thutupalli , and J. W. Shaevitz , “ Self-driven phase transitions drive *Myxococcus xanthus* fruiting body formation,” Phys. Rev. Lett. 122, 248102 (2019). 10.1103/PhysRevLett.122.248102 31322369

[12] S. Yazdi and A. M. Ardekani , “ Bacterial aggregation and biofilm formation in a vortical flow,” Biomicrofluidics 6, 044114 (2012). 10.1063/1.4771407 24339847 PMC3555698

[13] G. O'Toole , H. B. Kaplan , and R. Kolter , “ Biofilm formation as microbial development,” Annu. Rev. Microbiol. 54, 49–79 (2000). 10.1146/annurev.micro.54.1.49 11018124

[14] J. Henrichsen , “ Bacterial surface translocation: A survey and a classification,” Bacteriol. Rev. 36, 478–503 (1972). 10.1128/br.36.4.478-503.1972 4631369 PMC408329

[15] N. Wadhwa and H. C. Berg , “ Bacterial motility: Machinery and mechanisms,” Nat. Rev. Microbiol. 20, 161–173 (2022). 10.1038/s41579-021-00626-4 34548639

[16] N. C. Darnton , L. Turner , S. Rojevsky , and H. C. Berg , “ On torque and tumbling in swimming *Escherichia coli*,” J. Bacteriol. 189, 1756–1764 (2007). 10.1128/JB.01501-06 17189361 PMC1855780

[17] M. C. Leake , J. H. Chandler , G. H. Wadhams , F. Bai , R. M. Berry , and J. P. Armitage , “ Stoichiometry and turnover in single, functioning membrane protein complexes,” Nature 443, 355–358 (2006). 10.1038/nature05135 16971952

[18] A. L. Nord , E. Gachon , R. Perez-Carrasco , J. A. Nirody , A. Barducci , R. M. Berry , and F. Pedaci , “ Catch bond drives stator mechanosensitivity in the bacterial flagellar motor,” Proc. Natl. Acad. Sci. U. S. A. 114, 12952–12957 (2017). 10.1073/pnas.1716002114 29183968 PMC5724282

[19] K. M. Blair , L. Turner , J. T. Winkelman , H. C. Berg , and D. B. Kearns , “ A molecular clutch disables flagella in the Bacillus subtilis biofilm,” Science 320, 1636–1638 (2008). 10.1126/science.1157877 18566286

[20] Y. Sowa and R. M. Berry , “ Bacterial flagellar motor,” Q. Rev. Biophys. 41, 103–132 (2008). 10.1017/S0033583508004691 18812014

[21] B. Maier and G. C. L. Wong , “ How bacteria use type IV pili machinery on surfaces,” Trends Microbiol. 23, 775–788 (2015). 10.1016/j.tim.2015.09.002 26497940

[22] R. Balagam , D. B. Litwin , F. Czerwinski , M. Sun , H. B. Kaplan , J. W. Shaevitz , and O. A. Igoshin , “ *Myxococcus xanthus* gliding motors are elastically coupled to the substrate as predicted by the focal adhesion model of gliding motility,” PLoS Comput. Biol. 10, e1003619 (2014). 10.1371/journal.pcbi.1003619 24810164 PMC4014417

[23] T. Hölscher and Á. T. Kovács , “ Sliding on the surface: Bacterial spreading without an active motor,” Environ. Microbiol. 19, 2537–2545 (2017). 10.1111/1462-2920.13741 28370801

[24] E. J. Pollitt and S. P. Diggle , “ Defining motility in the *Staphylococci*,” Cell. Mol. Life Sci. 74, 2943–2958 (2017). 10.1007/s00018-017-2507-z 28378043 PMC5501909

[25] Q. Zhang , D. Nguyen , J. S. B. Tai , X. Xu , J. Nijjer , X. Huang , Y. Li , and J. Yan , “ Mechanical resilience of biofilms toward environmental perturbations mediated by extracellular matrix,” Adv. Funct. Mater. 32, 2110699 (2022). 10.1002/adfm.202110699

[26] K. Kovach , M. Davis-Fields , Y. Irie , K. Jain , S. Doorwar , K. Vuong , N. Dhamani , K. Mohanty , A. Touhami , and V. D. Gordon , “ Evolutionary adaptations of biofilms infecting cystic fibrosis lungs promote mechanical toughness by adjusting polysaccharide production,” npj Biofilms Microbiomes 3, 1 (2017). 10.1038/s41522-016-0007-9 28649402 PMC5445605

[27] T. Shaw , M. Winston , C. J. Rupp , I. Klapper , and P. Stoodley , “ Commonality of elastic relaxation times in biofilms,” Phys. Rev. Lett. 93, 098102 (2004). 10.1103/PhysRevLett.93.098102 15447143

[28] P. Stoodley , R. Cargo , C. J. Rupp , S. Wilson , and I. Klapper , “ Biofilm material properties as related to shear-induced deformation and detachment phenomena,” J. Ind. Microbiol. Biotechnol. 29, 361–367 (2002). 10.1038/sj.jim.7000282 12483479

[29] Y. F. Dufrêne and A. Persat , “ Mechanomicrobiology: How bacteria sense and respond to forces,” Nat. Rev. Microbiol. 18, 227–240 (2020). 10.1038/s41579-019-0314-2 31959911

[30] S. L. Kuchma , A. E. Ballok , J. H. Merritt , J. H. Hammond , W. Lu , J. D. Rabinowitz , and G. A. O'Toole , “ Cyclic-di-GMP-mediated repression of swarming motility by *Pseudomonas aeruginosa*: The *pilY1* gene and its impact on surface-associated behaviors,” Am. Soc. Microbiol. 192, 2950–2964 (2010). 10.1128/JB.01642-09 PMC2901681 20233936

[31] A. Siryaporn , S. L. Kuchma , G. A. O'Toole , and Z. Gitai , “ Surface attachment induces *Pseudomonas aeruginosa* virulence,” Proc. Natl. Acad. Sci. U. S. A. 111, 16860–16865 (2014). 10.1073/pnas.1415712111 25385640 PMC4250119

[32] A. Persat , Y. F. Inclan , J. N. Engel , H. A. Stone , and Z. Gitai , “ Type IV pili mechanochemically regulate virulence factors in *Pseudomonas aeruginosa*,” Proc. Natl. Acad. Sci. U. S. A. 112, 7563–7568 (2015). 10.1073/pnas.1502025112 26041805 PMC4475988

[33] B. J. Laventie , M. Sangermani , F. Estermann , P. Manfredi , R. Planes , I. Hug , T. Jaeger , E. Meunier , P. Broz , and U. Jenal , “ A surface-induced asymmetric program promotes tissue colonization by *Pseudomonas aeruginosa*,” Cell Host Microbe 25, 140–152.e6 (2019). 10.1016/j.chom.2018.11.008 30581112

[34] I. Hug , S. Deshpande , K. S. Sprecher , T. Pfohl , and U. Jenal , “ Second messenger–mediated tactile response by a bacterial rotary motor,” Science 358, 531–534 (2017). 10.1126/science.aan5353 29074777

[35] C. K. Ellison , J. Kan , R. S. Dillard , D. T. Kysela , A. Ducret , C. Berne , C. M. Hampton , Z. Ke , E. R. Wright , N. Biais *et al.*, “ Obstruction of pilus retraction stimulates bacterial surface sensing,” Science 358, 535–538 (2017). 10.1126/science.aan5706 29074778 PMC5805138

[36] L. S. Cairns , V. L. Marlow , E. Bissett , A. Ostrowski , and N. R. Stanley-Wall , “ A mechanical signal transmitted by the flagellum controls signalling in *Bacillus subtilis*,” Mol. Microbiol. 90, 6–21 (2013). 10.1111/mmi.12342 23888912 PMC3963450

[37] S. L. Kuchma , N. J. Delalez , L. M. Filkins , E. A. Snavely , J. P. Armitage , and G. A. O'Toole , “ Cyclic di-GMP-mediated repression of swarming motility by *Pseudomonas aeruginosa* PA14 requires the MotAB stator,” J. Bacteriol. 197, 420–430 (2015). 10.1128/JB.02130-14 25349157 PMC4285984

[38] S. Merino , J. G. Shaw , and J. M. Tomás , “ Bacterial lateral flagella: An inducible flagella system,” FEMS Microbiol. Lett. 263, 127–135 (2006). 10.1111/j.1574-6968.2006.00403.x 16978346

[39] E. A. Eloe , F. M. Lauro , R. F. Vogel , and D. H. Bartlett , “ The deep-sea bacterium *Photobacterium profundum* SS9 utilizes separate flagellar systems for swimming and swarming under high-pressure conditions,” Appl. Environ. Microbiol. 74, 6298–6305 (2008). 10.1128/AEM.01316-08 18723648 PMC2570297

[40] F. Wang , X. Xiao , H.-Y. Ou , Y. Gai , and F. Wang , “ Role and regulation of fatty acid biosynthesis in the response of *Shewanella piezotolerans* WP3 to different temperatures and pressures,” J. Bacteriol. 191, 2574–2584 (2009). 10.1128/JB.00498-08 19201790 PMC2668426

[41] K. Otto and T. J. Silhavy , “ Surface sensing and adhesion of *Escherichia coli* controlled by the Cpx-signaling pathway,” Proc. Natl. Acad. Sci. U. S. A. 99, 2287–2292 (2002). 10.1073/pnas.042521699 11830644 PMC122357

[42] N. Ruiz and T. J. Silhavy , “ Sensing external stress: Watchdogs of the *Escherichia coli* cell envelope,” Curr. Opin. Microbiol. 8, 122–126 (2005). 10.1016/j.mib.2005.02.013 15802241

[43] J. P. Nataro and J. B. Kaper , “ Diarrheagenic *Escherichia coli*,” Clin. Microbiol. Rev. 11, 142–201 (1998). 10.1128/CMR.11.1.142 9457432 PMC121379

[44] T. Shimizu , K. Ichimura , and M. Noda , “ The surface sensor NlpE of enterohemorrhagic *Escherichia coli* contributes to regulation of the type III secretion system and flagella by the Cpx response to adhesion,” Infect. Immun. 84, 537–549 (2016). 10.1128/IAI.00881-15 26644384 PMC4730559

[45] G. Alsharif , S. Ahmad , M. S. Islam , R. Shah , S. J. Busby , and A. M. Krachler , “ Host attachment and fluid shear are integrated into a mechanical signal regulating virulence in *Escherichia coli* O157: H7,” Proc. Natl. Acad. Sci. U. S. A. 112, 5503–5508 (2015). 10.1073/pnas.1422986112 25870295 PMC4418854

[46] E. K. Chu , O. Kilic , H. Cho , A. Groisman , and A. Levchenko , “ Self-induced mechanical stress can trigger biofilm formation in uropathogenic *Escherichia coli*,” Nat. Commun. 9(1), 4087 (2018). 10.1038/s41467-018-06552-z 30291231 PMC6173693

[47] J. C. Fong , K. Karplus , G. K. Schoolnik , and F. H. Yildiz , “ Identification and characterization of RbmA, a novel protein required for the development of rugose colony morphology and biofilm structure in *Vibrio cholerae*,” J. Bacteriol. 188, 1049–1059 (2006). 10.1128/JB.188.3.1049-1059.2006 16428409 PMC1347326

[48] C. Absalon , K. Van Dellen , and P. I. Watnick , “ A communal bacterial adhesin anchors biofilm and bystander cells to surfaces,” PLoS Pathogens 7, e1002210 (2011). 10.1371/journal.ppat.1002210 21901100 PMC3161981

[49] O. Lieleg , M. Caldara , R. Baumgärtel , and K. Ribbeck , “ Mechanical robustness of *Pseudomonas aeruginosa* biofilms,” Soft Matter 7, 3307–3314 (2011). 10.1039/c0sm01467b 21760831 PMC3134232

[50] J. Yan , A. Moreau , S. Khodaparast , A. Perazzo , J. Feng , C. Fei , S. Mao , S. Mukherjee , A. Košmrlj , N. S. Wingreen *et al.*, “ Bacterial biofilm material properties enable removal and transfer by capillary peeling,” Adv. Mater. 30, 1804153 (2018). 10.1002/adma.201804153 PMC8865467 30368924

[51] T. G. Mason , K. Ganesan , J. H. van Zanten , D. Wirtz , and S. C. Kuo , “ Particle tracking microrheology of complex fluids,” Phys. Rev. Lett. 79, 3282–3285 (1997). 10.1103/PhysRevLett.79.3282

[52] J. Liu , M. L. Gardel , K. Kroy , E. Frey , B. D. Hoffman , J. C. Crocker , A. R. Bausch , and D. A. Weitz , “ Microrheology probes length scale dependent rheology,” Phys. Rev. Lett. 96, 118104 (2006). 10.1103/PhysRevLett.96.118104 16605878

[53] R. M. Robertson-Anderson , “ Optical tweezers microrheology: From the basics to advanced techniques and applications,” ACS Macro Lett. 7, 968–975 (2018). 10.1021/acsmacrolett.8b00498 35650960 PMC9163451

[54] S. S. Rogers , C. Van Der Walle , and T. A. Waigh , “ Microrheology of bacterial biofilms in vitro: *Staphylococcus aureus* and *Pseudomonas aeruginosa*,” Langmuir 24, 13549–13555 (2008). 10.1021/la802442d 18980352

[55] S. C. Chew , B. Kundukad , T. Seviour , J. R. Van der Maarel , L. Yang , S. A. Rice , P. Doyle , and S. Kjelleberg , “ Dynamic remodeling of microbial biofilms by functionally distinct exopolysaccharides,” MBio 5, e01536-01514 (2014). 10.1128/mBio.01536-14 25096883 PMC4128364

[56] A. Birjiniuk , N. Billings , E. Nance , J. Hanes , K. Ribbeck , and P. S. Doyle , “ Single particle tracking reveals spatial and dynamic organization of the Escherichia coli biofilm matrix,” New J. Phys. 16, 085014 (2014). 10.1088/1367-2630/16/8/085014 PMC4234077 25414591

[57] M. Lekka , D. Gil , K. Pogoda , J. Dulińska-Litewka , R. Jach , J. Gostek , O. Klymenko , S. Prauzner-Bechcicki , Z. Stachura , J. Wiltowska-Zuber , K. Okoń , and P. Laidler , “ Cancer cell detection in tissue sections using AFM,” Arch. Biochem. Biophys. 518, 151–156 (2012). 10.1016/j.abb.2011.12.013 22209753

[58] J. W. Goss and C. B. Volle , “ Using atomic force microscopy to illuminate the biophysical properties of microbes,” ACS Appl. Bio Mater. 3, 143–155 (2019). 10.1021/acsabm.9b00973 PMC7447269 32851362

[59] A. Razatos , “ Application of atomic force microscopy to study initial events of bacterial adhesion,” Methods Enzymol. 337, 276–285 (2001). 10.1016/S0076-6879(01)37021-0 11398436

[60] G. Zeng , T. Müller , and R. L. Meyer , “ Single-cell force spectroscopy of bacteria enabled by naturally derived proteins,” Langmuir 30, 4019–4025 (2014). 10.1021/la404673q 24654836

[61] E. Miller , T. Garcia , S. Hultgren , and A. F. Oberhauser , “ The mechanical properties of *E. coli* type 1 pili measured by atomic force microscopy techniques,” Biophys. J. 91, 3848–3856 (2006). 10.1529/biophysj.106.088989 16950852 PMC1630485

[62] M. Forero , O. Yakovenko , E. V. Sokurenko , W. E. Thomas , and V. Vogel , “ Uncoiling mechanics of *Escherichia coli* type I fimbriae are optimized for catch bonds,” PLoS Biol. 4, e298 (2006). 10.1371/journal.pbio.0040298 16933977 PMC1557399

[63] L. M. Nilsson , W. E. Thomas , E. Trintchina , V. Vogel , and E. V. Sokurenko , “ Catch bond-mediated adhesion without a shear threshold: *Trimannose versus monomannose interactions with the FimH adhesin of Escherichia coli*,” J. Biol. Chem. 281, 16656–16663 (2006). 10.1074/jbc.M511496200 16624825

[64] W. M. Weaver , S. Dharmaraja , V. Milisavljevic , and D. Di Carlo , “ The effects of shear stress on isolated receptor-ligand interactions of *Staphylococcus epidermidis* and human plasma fibrinogen using molecularly patterned microfluidics,” Lab Chip 11, 883–889 (2011). 10.1039/c0lc00414f 21249255

[65] K. I. Pappelbaum , C. Gorzelanny , S. Grässle , J. Suckau , M. W. Laschke , M. Bischoff , C. Bauer , M. Schorpp-Kistner , C. Weidenmaier , R. Schneppenheim *et al.*, “ Ultralarge von Willebrand factor fibers mediate luminal *Staphylococcus aureus* adhesion to an intact endothelial cell layer under shear stress,” Circulation 128, 50–59 (2013). 10.1161/CIRCULATIONAHA.113.002008 23720451

[66] R. Hartmann , P. K. Singh , P. Pearce , R. Mok , B. Song , F. Díaz-Pascual , J. Dunkel , and K. Drescher , “ Emergence of three-dimensional order and structure in growing biofilms,” Nat. Phys. 15, 251–256 (2019). 10.1038/s41567-018-0356-9 31156716 PMC6544526

[67] K. Drescher , Y. Shen , B. L. Bassler , and H. A. Stone , “ Biofilm streamers cause catastrophic disruption of flow with consequences for environmental and medical systems,” Proc. Natl. Acad. Sci. U. S. A. 110, 4345–4350 (2013). 10.1073/pnas.1300321110 23401501 PMC3600445

[68] C. J. Rupp , C. A. Fux , and P. Stoodley , “ Viscoelasticity of *Staphylococcus aureus* biofilms in response to fluid shear allows resistance to detachment and facilitates rolling migration,” Appl. Environ. Microbiol. 71, 2175–2178 (2005). 10.1128/AEM.71.4.2175-2178.2005 15812054 PMC1082509

[69] J. E. Sanfilippo , A. Lorestani , M. D. Koch , B. P. Bratton , A. Siryaporn , H. A. Stone , and Z. Gitai , “ Microfluidic-based transcriptomics reveal force-independent bacterial rheosensing,” Nat. Microbiol. 4, 1274–1281 (2019). 10.1038/s41564-019-0455-0 31086313 PMC6656604

[70] A. P. Hitchens and M. C. Leikind , “ The introduction of agar-agar into bacteriology,” J. Bacteriol. 37, 485–493 (1939). 10.1128/jb.37.5.485-493.1939 16560221 PMC374482

[71] K. Little , J. Austerman , J. Zheng , K. A. Gibbs , and G. O'Toole , “ Cell shape and population migration are distinct steps of *Proteus mirabilis* swarming that are decoupled on high-percentage agar,” J. Bacteriol. 201, e00726-00718 (2019). 10.1128/JB.00726-18 30858303 PMC6509654

[72] J. Yan , C. D. Nadell , H. A. Stone , N. S. Wingreen , and B. L. Bassler , “ Extracellular-matrix-mediated osmotic pressure drives *Vibrio cholerae* biofilm expansion and cheater exclusion,” Nat. Commun. 8, 327 (2017). 10.1038/s41467-017-00401-1 28835649 PMC5569112

[73] K. Kalai Chelvam , L. C. Chai , and K. L. Thong , “ Variations in motility and biofilm formation of *Salmonella enterica* serovar Typhi,” Gut Pathogens 6(1), 2 (2014). 10.1186/1757-4749-6-2 24499680 PMC3922113

[74] S. Srinivasan , C. N. Kaplan , and L. Mahadevan , “ A multiphase theory for spreading microbial swarms and films,” eLife 8, e42697 (2019). 10.7554/eLife.42697 31038122 PMC6491038

[75] H. H. Tuson , L. D. Renner , and D. B. Weibel , “ Polyacrylamide hydrogels as substrates for studying bacteria,” Chem. Commun. 48, 1595–1597 (2012). 10.1039/C1CC14705F PMC3705218 22039586

[76] M. E. Asp , M.-T. Ho Thanh , D. A. Germann , R. J. Carroll , A. Franceski , R. D. Welch , A. Gopinath , and A. E. Patteson , “ Spreading rates of bacterial colonies depend on substrate stiffness and permeability,” PNAS Nexus 1, pgac025 (2022). 10.1093/pnasnexus/pgac025 36712798 PMC9802340

[77] A. Cont , T. Rossy , Z. Al-Mayyah , and A. Persat , “ Biofilms deform soft surfaces and disrupt epithelia,” eLife 9, e56533 (2020). 10.7554/eLife.56533 33025904 PMC7556879

[78] K. W. Kolewe , J. Zhu , N. R. Mako , S. S. Nonnenmann , and J. D. Schiffman , “ Bacterial adhesion is affected by the thickness and stiffness of poly (ethylene glycol) hydrogels,” ACS Appl. Mater. Interfaces 10, 2275–2281 (2018). 10.1021/acsami.7b12145 29283244 PMC5785418

[79] F. Song , H. Wang , K. Sauer , and D. Ren , “ Cyclic-di-GMP and *oprF* are involved in the response of *Pseudomonas aeruginosa* to substrate material stiffness during attachment on polydimethylsiloxane (PDMS),” Front. Microbiol. 9, 110 (2018). 10.3389/fmicb.2018.00110 29449837 PMC5799285

[80] F. Song and D. Ren , “ Stiffness of cross-linked poly (dimethylsiloxane) affects bacterial adhesion and antibiotic susceptibility of attached cells,” Langmuir 30, 10354–10362 (2014). 10.1021/la502029f 25117376

[81] B. Sabass , M. D. Koch , G. Liu , H. A. Stone , and J. W. Shaevitz , “ Force generation by groups of migrating bacteria,” Proc. Natl. Acad. Sci. U. S. A. 114, 7266–7271 (2017). 10.1073/pnas.1621469114 28655845 PMC5514709

[82] M. Dembo and Y.-L. Wang , “ Stresses at the cell-to-substrate interface during locomotion of fibroblasts,” Biophys. J. 76, 2307–2316 (1999). 10.1016/S0006-3495(99)77386-8 10096925 PMC1300203

[83] C. E. Kandow , P. C. Georges , P. A. Janmey , and K. A. Beningo , “ Polyacrylamide hydrogels for cell mechanics: Steps toward optimization and alternative uses,” Methods Cell Biol. 83, 29–46 (2007). 10.1016/S0091-679X(07)83002-0 17613303

[84] L. D. Landau , E. M. Lifšic , E. M. Lifshitz , A. M. Kosevich , and L. P. Pitaevskii , *Theory of Elasticity* ( Elsevier, 1986), Vol. 7.

[85] M.-C. Duvernoy , T. Mora , M. Ardré , V. Croquette , D. Bensimon , C. Quilliet , J.-M. Ghigo , M. Balland , C. Beloin , S. Lecuyer , and N. Desprat , “ Asymmetric adhesion of rod-shaped bacteria controls microcolony morphogenesis,” Nat. Commun. 9, 1120 (2018). 10.1038/s41467-018-03446-y 29549338 PMC5856753

[86] P. K. Sahoo , R. Janissen , M. P. Monteiro , A. Cavalli , D. M. Murillo , M. V. Merfa , C. L. Cesar , H. F. Carvalho , A. A. De Souza , E. P. A. M. Bakkers , and M. A. Cotta , “ Nanowire arrays as cell force sensors to investigate adhesin-enhanced holdfast of single cell bacteria and biofilm stability,” Nano Lett. 16, 4656–4664 (2016). 10.1021/acs.nanolett.6b01998 27336224

[87] S. Zheng , M. Bawazir , A. Dhall , H.-E. Kim , L. He , J. Heo , and G. Hwang , “ Implication of surface properties, bacterial motility, and hydrodynamic conditions on bacterial surface sensing and their initial adhesion,” Front. Bioeng. Biotechnol. 9, 643722 (2021). 10.3389/fbioe.2021.643722 33644027 PMC7907602

[88] I. Yoda , H. Koseki , M. Tomita , T. Shida , H. Horiuchi , H. Sakoda , and M. Osaki , “ Effect of surface roughness of biomaterials on *Staphylococcus epidermidis* adhesion,” BMC Microbiol. 14, 234 (2014). 10.1186/s12866-014-0234-2 25179448 PMC4161769

[89] R. Xing , S. P. Lyngstadaas , J. E. Ellingsen , S. Taxt-Lamolle , and H. J. Haugen , “ The influence of surface nanoroughness, texture and chemistry of TiZr implant abutment on oral biofilm accumulation,” Clin. Oral Implants Res. 26, 649–656 (2015). 10.1111/clr.12354 25906328

[90] S. Perni and P. Prokopovich , “ Micropatterning with conical features can control bacterial adhesion on silicone,” Soft Matter 9, 1844–1851 (2013). 10.1039/C2SM26828K

[91] L. Rizzello , B. Sorce , S. Sabella , G. Vecchio , A. Galeone , V. Brunetti , R. Cingolani , and P. P. Pompa , “ Impact of nanoscale topography on genomics and proteomics of adherent bacteria,” Acs Nano 5, 1865–1876 (2011). 10.1021/nn102692m 21344880

[92] A. K. Epstein , D. Hong , P. Kim , and J. Aizenberg , “ Biofilm attachment reduction on bioinspired, dynamic, micro-wrinkling surfaces,” New J. Phys. 15, 095018 (2013). 10.1088/1367-2630/15/9/095018

[93] S. W. Lee , J. Carnicelli , D. Getya , I. Gitsov , K. S. Phillips , and D. Ren , “ Biofilm removal by reversible shape recovery of the substrate,” ACS Appl. Mater. Interfaces 13, 17174–17182 (2021). 10.1021/acsami.0c20697 33822590 PMC8153534

[94] P. A. Levin and E. R. Angert , “ Small but mighty: Cell size and bacteria,” Cold Spring Harbor Perspect. Biol. 7, a019216 (2015). 10.1101/cshperspect.a019216 PMC4484965 26054743

[95] T. J. Beveridge , “ Structures of gram-negative cell walls and their derived membrane vesicles,” J. Bacteriol. 181, 4725–4733 (1999). 10.1128/JB.181.16.4725-4733.1999 10438737 PMC93954

[96] R. M. Macnab , “ Examination of bacterial flagellation by dark-field microscopy,” J. Clin. Microbiol. 4, 258–265 (1976). 10.1128/jcm.4.3.258-265.1976 823174 PMC274447

[97] H. P. Erickson , D. E. Anderson , and M. Osawa , “ FtsZ in bacterial cytokinesis: Cytoskeleton and force generator all in one,” Microbiol. Mol. Biol. Rev. 74, 504–528 (2010). 10.1128/MMBR.00021-10 21119015 PMC3008173

[98] L. A. Genova , M. F. Roberts , Y.-C. Wong , C. E. Harper , A. G. Santiago , B. Fu , A. Srivastava , W. Jung , L. M. Wang , Ł. Krzemiński , X. Mao , X. Sun , C.-Y. Hui , P. Chen , and C. J. Hernandez , “ Mechanical stress compromises multicomponent efflux complexes in bacteria,” Proc. Natl. Acad. Sci. 116, 25462–25467 (2019). 10.1073/pnas.1909562116 31772020 PMC6925999

[99] F. Depardieu , I. Podglajen , R. Leclercq , E. Collatz , and P. Courvalin , “ Modes and modulations of antibiotic resistance gene expression,” Clin. Microbiol. Rev. 20, 79–114 (2007). 10.1128/CMR.00015-06 17223624 PMC1797629

[100] M. D. Koch , M. E. Black , E. Han , J. W. Shaevitz , and Z. Gitai , “ *Pseudomonas aeruginosa* distinguishes surfaces by stiffness using retraction of type IV pili,” Proc. Natl. Acad. Sci. 119, e2119434119 (2022). 10.1073/pnas.2119434119 35561220 PMC9171759

[101] J. McCutcheon and G. Southam , “ Advanced biofilm staining techniques for TEM and SEM in geomicrobiology: Implications for visualizing EPS architecture, mineral nucleation, and microfossil generation,” Chem. Geol. 498, 115–127 (2018). 10.1016/j.chemgeo.2018.09.016

[102] R. P. Dassanayake , S. M. Falkenberg , J. A. Stasko , A. L. Shircliff , J. D. Lippolis , and R. E. Briggs , “ Identification of a reliable fixative solution to preserve the complex architecture of bacterial biofilms for scanning electron microscopy evaluation,” PLoS One 15, e0233973 (2020). 10.1371/journal.pone.0233973 32470063 PMC7259777

[103] M. Lu , T. Dai , C. K. Murray , and M. X. Wu , “ bactericidal property of oregano oil against multidrug-resistant clinical isolates,” Front. Microbiol. 9, 2329 (2018). 10.3389/fmicb.2018.02329 30344513 PMC6182053

[104] A. M. T. Barnes , K. S. Ballering , R. S. Leibman , C. L. Wells , and G. M. Dunny , “ Enterococcus faecalis Produces abundant extracellular structures containing DNA in the absence of cell lysis during early biofilm formation,” mBio 3, e00193 (2012). 10.1128/mBio.00193-12 22829679 PMC3413405

[105] P. Bravo , S. L. Ng , K. A. MacGillivray , B. K. Hammer , and P. J. Yunker , “ Vertical growth dynamics of biofilms,” Proc. Natl. Acad. Sci. 120(11), e2214211120 (2023). 36881625 10.1073/pnas.2214211120 PMC10089195

[106] A. Kalziqi , D. Yanni , J. Thomas , S. L. Ng , S. Vivek , B. K. Hammer , and P. J. Yunker , “ Immotile active matter: Activity from death and reproduction,” Phys. Rev. Lett. 120, 018101 (2018). 10.1103/PhysRevLett.120.018101 29350941

[107] F. Jacob and J. Monod , “ Genetic regulatory mechanisms in the synthesis of proteins,” J. Mol. Biol. 3, 318–356 (1961). 10.1016/S0022-2836(61)80072-7 13718526

[108] C. Pinto , R. Melo-Miranda , I. Gordo , and A. Sousa , “ The selective advantage of the lac operon for *Escherichia coli* is conditional on diet and microbiota composition,” Front. Microbiol. 12, 2042 (2021). 10.3389/fmicb.2021.709259 PMC8333865 34367115

[109] A. Mogk , T. Tomoyasu , P. Goloubinoff , S. Rüdiger , D. Röder , H. Langen , and B. Bukau , “ Identification of thermolabile *Escherichia coli* proteins: Prevention and reversion of aggregation by DnaK and ClpB,” EMBO J. 18, 6934–6949 (1999). 10.1093/emboj/18.24.6934 10601016 PMC1171757

[110] D. Roncarati and V. Scarlato , “ Regulation of heat-shock genes in bacteria: From signal sensing to gene expression output,” FEMS Microbiol. Rev. 41, 549–574 (2017). 10.1093/femsre/fux015 28402413

[111] Q. Wang , J. G. Frye , M. McClelland , and R. M. Harshey , “ Gene expression patterns during swarming in Salmonella typhimurium: Genes specific to surface growth and putative new motility and pathogenicity genes,” Mol. Microbiol. 52, 169–187 (2004). 10.1111/j.1365-2958.2003.03977.x 15049819

[112] D. J. P. G. Rocha , T. L. P. Castro , E. R. G. R. Aguiar , and L. G. C. Pacheco , *Gene Expression Analysis in Bacteria by RT-qPCR BT - Quantitative Real-Time PCR: Methods and Protocols*, edited by R. Biassoni and A. Raso ( Springer, New York, NY, 2020), pp. 119–137. 10.1007/978-1-4939-9833-3_10 31578692

[113] H. K. Tiong and P. M. Muriana , “ RT-qPCR analysis of 15 genes encoding putative surface proteins involved in adherence of Listeria monocytogenes,” Pathogens 5, 60 (2016). 10.3390/pathogens5040060 27706070 PMC5198160

[114] G. S. Lorite , R. Janissen , J. H. Clerici , C. M. Rodrigues , J. P. Tomaz , B. Mizaikoff , C. Kranz , A. A. de Souza , and M. A. Cotta , “ Surface physicochemical properties at the micro and nano length scales: Role on bacterial adhesion and Xylella fastidiosa biofilm development,” PLoS One 8, e75247 (2013). 10.1371/journal.pone.0075247 24073256 PMC3779164

[115] N. J. Croucher and N. R. Thomson , “ Studying bacterial transcriptomes using RNA-seq,” Curr. Opin. Microbiol. 13, 619–624 (2010). 10.1016/j.mib.2010.09.009 20888288 PMC3025319

[116] C. J. Jones , N. Grotewold , D. J. Wozniak , and E. S. Gloag , “ *Pseudomonas aeruginosa* initiates a rapid and specific transcriptional response during surface attachment,” J. Bacteriology 204, e00086-22 (2022). 10.1128/jb.00086-22 PMC9112911 35467391

[117] K. Avican , J. Aldahdooh , M. Togninalli , A. K. M. F. Mahmud , J. Tang , K. M. Borgwardt , M. Rhen , and M. Fällman , “ RNA atlas of human bacterial pathogens uncovers stress dynamics linked to infection,” Nat. Commun. 12, 3282 (2021). 10.1038/s41467-021-23588-w 34078900 PMC8172932

[118] Z. Li , M. Xu , H. Wei , L. Wang , and M. Deng , “ RNA–seq analyses of antibiotic resistance mechanisms in Serratia marcescens,” Mol. Med. Rep. 20, 745–754 (2019). 10.3892/mmr.2019.10281 31180518 PMC6580034

[119] J. Briffotaux , S. Liu , and B. Gicquel , “ Genome-wide transcriptional responses of mycobacterium to antibiotics,” Front. Microbiol. 10, 249 (2019). 30842759 10.3389/fmicb.2019.00249 PMC6391361

[120] M. Tata , M. T. Wolfinger , F. Amman , N. Roschanski , A. Dötsch , E. Sonnleitner , S. Häussler , and U. Bläsi , “ RNASeq based transcriptional profiling of *Pseudomonas aeruginosa* PA14 after short- and long-term anoxic cultivation in synthetic cystic fibrosis sputum medium,” PLoS One 11, e0147811 (2016). 10.1371/journal.pone.0147811 26821182 PMC4731081

[121] A. Otto , D. Becher , and F. Schmidt , “ Quantitative proteomics in the field of microbiology,” Proteomics 14, 547–565 (2014). 10.1002/pmic.201300403 24376008

[122] F. J. Pérez-Llarena and G. Bou , “ Proteomics as a tool for studying bacterial virulence and antimicrobial resistance,” Front. Microbiol. 7, 410–410 (2016). 10.3389/fmicb.2016.00410 27065974 PMC4814472

[123] H. Aschard , B. J. Vilhjálmsson , N. Greliche , P. E. Morange , D. A. Trégouët , and P. Kraft , “ Maximizing the power of principal-component analysis of correlated phenotypes in genome-wide association studies,” Am. J. Human Genet. 94, 662–676 (2014). 10.1016/j.ajhg.2014.03.016 24746957 PMC4067564

[124] J. A. Comstock , M. E. Asp , F. Bahar , I. Lee , A. E. Patteson , and R. D. Welch , “ Phenotypic similarity is a measure of functional redundancy within homologous gene families,” bioRxiv (2022).

[125] H. Shi , A. Colavin , M. Bigos , C. Tropini , R. D. Monds , and K. C. Huang , “ Deep phenotypic mapping of bacterial cytoskeletal mutants reveals physiological robustness to cell size,” Curr. Biol. 27, 3419–3429 (2017). 10.1016/j.cub.2017.09.065 29103935

[126] S. Sridhar , S. Forrest , B. Warne , M. Maes , S. Baker , G. Dougan , and J. Bartholdson Scott , “ High-content imaging to phenotype antimicrobial effects on individual bacteria at scale,” mSystems 6, e00028-21 (2021). 34006623 10.1128/mSystems.00028-21 PMC8269201

[127] W. S. Noble , “ What is a support vector machine?,” Nat. Biotechnol. 24(12), 1565–1567 (2006). 17160063 10.1038/nbt1206-1565

[128] M. F. Z. Wang and R. Fernandez-Gonzalez , “ (Machine-)learning to analyze *in vivo* microscopy: Support vector machines,” Biochim. Biophys. Acta, Protein Proteomics 2017, 1719–1727 (2017). 10.1016/j.bbapap.2017.09.013 28974388

[129] S. Panigrahi , D. Murat , A. Le Gall , E. Martineau , K. Goldlust , J.-B. Fiche , S. Rombouts , M. Nöllmann , L. Espinosa , and T. Mignot , “ Misic, a general deep learning-based method for the high-throughput cell segmentation of complex bacterial communities,” eLife 10, e65151 (2021). 34498586 10.7554/eLife.65151 PMC8478410

[130] J. B. Lugagne , H. Lin , and M. J. Dunlop , “ Delta: Automated cell segmentation, tracking, and lineage reconstruction using deep learning,” PLoS Comput. Biol. 16, e1007673 (2020). 32282792 10.1371/journal.pcbi.1007673 PMC7153852

[131] A. O. Basile and M. D. R. Ritchie , “ Informatics and machine learning to define the phenotype,” in *Expert Review of Molecular Diagnostics* ( Taylor and Francis Ltd, 2018). 10.1080/14737159.2018.1439380 PMC6080627 29431517

[132] A. Drouin , G. Letarte , F. Raymond , M. Marchand , J. Corbeil , and F. Laviolette , “ Interpretable genotype-to-phenotype classifiers with performance guarantees,” Sci. Rep. 9, 4071 (2019). 30858411 10.1038/s41598-019-40561-2 PMC6411721

[133] J. P. Allen , E. Snitkin , N. B. Pincus , and A. R. Hauser , “ Forest and trees: Exploring bacterial virulence with genome-wide association studies and machine learning,” in *Trends in Microbiology* ( Elsevier, 2021). 10.1016/j.tim.2020.12.002 PMC8187264 33455849

[134] M. K. Transtrum and P. Qiu , “ Model reduction by manifold boundaries,” Phys. Rev. Lett. 113, 098701 (2014). 10.1103/PhysRevLett.113.098701 25216014 PMC4425275

[135] D. G. Whittaker , J. Wang , J. G. Shuttleworth , R. Venkateshappa , J. M. Kemp , T. W. Claydon , and G. R. Mirams , “ Ion channel model reduction using manifold boundaries,” J. R. Soc. Interface 19, 20220193 (2022). 35946166 10.1098/rsif.2022.0193 PMC9363999

[136] N. Verstraeten , K. Braeken , B. Debkumari , M. Fauvart , J. Fransaer , J. Vermant , and J. Michiels , “ Living on a surface: Swarming and biofilm formation,” Trends Microbiol. 16, 496–506 (2008). 10.1016/j.tim.2008.07.004 18775660

[137] Y. Zhao , F. Song , H. Wang , J. Zhou , and D. Ren , “ Phagocytosis of *Escherichia coli* biofilm cells with different aspect ratios: A role of substratum material stiffness,” Appl. Microbiol. Biotechnol. 101, 6473–6481 (2017). 10.1007/s00253-017-8394-2 28707067 PMC5624331

[138] X. Liu , K. Zhu , X. Duan , P. Wang , Y. Han , W. Peng , and J. Huang , “ Extracellular matrix stiffness modulates host-bacteria interactions and antibiotic therapy of bacterial internalization,” Biomaterials 277, 121098 (2021). 10.1016/j.biomaterials.2021.121098 34478931

[139] X. Pierrat , J. P. H. Wong , Z. Al-Mayyah , and A. Persat , “ The mammalian membrane microenvironment regulates the sequential attachment of bacteria to host cells,” mBio 12, e01392-01321 (2021). 10.1128/mBio.01392-21 34340544 PMC8406306

[140] E. E. Bastounis , Y.-T. Yeh , and J. A. Theriot , “ Matrix stiffness modulates infection of endothelial cells by Listeria monocytogenes via expression of cell surface vimentin,” Mol. Biol. Cell 29, 1571–1589 (2018). 10.1091/mbc.E18-04-0228 29718765 PMC6080647

[141] S. Moorthy , F. J. Byfield , P. A. Janmey , and E. A. Klein , “ Matrix stiffness regulates endosomal escape of uropathogenic *E. coli*,” Cell. Microbiol. 22, e13196 (2020). 10.1111/cmi.13196 32083802

[142] Q. Hou , A. Keren-Paz , E. Korenblum , R. Oved , S. Malitsky , and I. Kolodkin-Gal , “ Weaponizing volatiles to inhibit competitor biofilms from a distance,” npj Biofilms Microbiomes 7(2), 1–8 (2021). 33402677 10.1038/s41522-020-00174-4 PMC7785731

